# Substantive Morphological Descriptions, Phylogenetic Analysis and Single Nucleotide Polymorphisms of *Aspergillus* Species From *Foeniculum vulgare*

**DOI:** 10.3389/fmicb.2022.832320

**Published:** 2022-02-18

**Authors:** Pranab Kumar Mahata, Regina Sharmila Dass, Archana Pan, Babylakshmi Muthusamy

**Affiliations:** ^1^Fungal Genetics and Mycotoxicology Laboratory, Department of Microbiology, School of Life Sciences, Pondicherry University, Pondicherry, India; ^2^Centre for Bioinformatics, School of Life Sciences, Pondicherry University, Pondicherry, India; ^3^Institute of Bioinformatics, International Tech Park, Bengaluru, India; ^4^Manipal Academy of Higher Education, Manipal, India

**Keywords:** fungal genomics, *Aspergillus*, polyphasic, phylogenetic analysis, single nucleotide polymorphisms (SNPs), single nucleotide variations (SNVs), non-synonymous and synonymous mutation

## Abstract

Ascomycetous fungi are found associated with a wide variety of substrates which range from fresh water to marine ecosystems, tropical to temperate forest soils and deserts, throughout the world over. These demystifying fungi exist as endophytes, pathogens and saprobes. They have been studied due to their ability to contaminate foods and feedstuffs, causing an elaboration of mycotoxins. The objectives of the study included extensive analyses of the morphological features of fungi, especially *Aspergilli*, which have been presented while studying them on specific mycological media. It is also an elaborate compilation of substantive macro- and micro-morphological characterization of different *Aspergilli* isolated from the spice *Foeniculum vulgare* used in India and other countries in the world. Further, a first of its kind attempt has been made to study their relative abundance and frequency of occurrence, molecular phylogeny and genetic relatedness to characterize the *Aspergilli* into specific sections, groups and clades. Single nucleotide polymorphism (SNP) analysis was carried out to evaluate the functional consequences of nucleotide variations, synonymous and non-synonymous mutations in the protein structure. The study resulted in a total of 3,506 *Aspergillus* isolates, which were obtained from seventy (70) fennel samples, representing 14 *Aspergillus* species. The two most frequently found species were *A. niger* and *A. flavus* with a relative abundance of 32.24 and 11.63%, respectively. The taxonomy and current placements have been reappraised with suggestions and prospects for future research from six sections namely *Terrei, Flavi*, *Fumigati*, *Nidulantes*, *Nigri*, and *Versicolores*. In addition, a total number of 27 isolates were studied and deposited at the National Centre for Biotechnology Information (NCBI) and five *Aspergillus* species have been identified and are being reported for the first time from the fennel seeds, based on partial sequence analysis of the official fungal barcode namely, Internal Transcribed Spacer (ITS) and a functional gene, beta tubulin gene locus, coupled with phenotypic characterization. SNPs for specific DNA regions have been used to identify variants in *Aspergilli* obtained from Indian fennel seeds for the first time. The need for a polyphasic approach of morphological identification and genetic characterization of *Aspergilli* from *Foeniculum vulgare* is addressed and presented here in adequate detail. Our current work makes extensive use of partial beta-tubulin gene sequences analyses to evaluate the association between SNPs in five *Aspergillus* species sections.

## Introduction

*Foeniculum vulgare* Mill, commonly known as fennel in English and *saunf* in Hindi, belongs to the Umbelliferae (Apiaceae) family, is a flowering plant species, and is accredited by the International Code of Botanical Nomenclature (ICBN) (Badgujar et al., 2014). It is a perennial type of herb and is available in many regions like the United States, Northern Europe, southern Canada, Asia and Australia. This spice is flavorful, aromatic with culinary and medicinal uses. Fennel seeds are considered as a rich source of protein (2%), dietary fiber (10%), vitamin A (4.5%), thiamin (1%), vitamin C (14%) and dietary minerals like calcium (4%), iron (4%), magnesium (4%), and manganese (7%) with essential fatty acids. They are known to possess properties like antioxidant, antitumor, cryoprotective, hyper protective, hyperglycemic, and estrogenic activities (Javidnia et al., 2003; Samadi-Noshahr et al., 2021). It has a memory enhancing effect and can reduce stress (Koppula and Kumar, 2013). More importantly, *F. vulgare* has been used in the control of infectious diseases of bacterial, viral, fungal, mycobacterial and protozoan origin (Rather et al., 2016).

Statistics reveal that fennel production in India has doubled (58,265 tons) ever since the year 2000–2001 (27,332 tons), indicating the consumption trend in the sub-continent. Fennel is not just produced and processed in India, but it also exported. India exported around 23,562,460, and 20,295,380 million metric tons in the years 2018 and 2020–2021, respectively (Anonymous, 2020). The Ministry of Food Processing has estimated a loss of nearly 93 crores INR, due to harvest and post-harvest losses of India’s agricultural products (Moloney, 2019), fennel being one of them.

*F. vulgare*, like most cereals and grains can be infected by range of mycoflora, some of which may significantly damage the economic value of the crop. *Aspergillus* spp. infection and several other genera have been of specific concern in the recent years, because these fungal phytopathogens produce toxic metabolites (mycotoxins) which represent significant contaminants of food (Ahmad et al., 2014), feed (Streit et al., 2012), and agricultural commodities, spices being one of them (Makhlouf et al., 2019). The genus *Aspergillus* consists of a few over one hundred mold species, ever since its first characterization nearly 300 years ago. It is mitosporic and conidial group of fungi, where in some species exhibit teleomorphic stages and hence are classified under the division Ascomycota (Bennett, 2010). Apart from their wide use in the industry for benefits (Casas López et al., 2004), *Aspergillus* species are prolific producers of secondary metabolites known as mycotoxins (Goto et al., 1996). Some species of *Aspergillus* cause diseases in humans (Alshehri and Palanisamy, 2020; Kashyap, 2020), animals and birds (Arné et al., 2021). More than sixty *Aspergillus* sp. are pathogens which pose a major health concern (Pal et al., 2014). *Aspergilli* are the common contaminants of food and feedstuffs. Spices (for example, fennel seeds) occupy a prominent role in the day-to-day culinary preparations in India (Siruguri and Bhat, 2015) and are not free from being contaminated by a variety of mycotoxins. Post-harvest contamination and spoilage during storage deteriorates the nutritive value, make them unfit for consumption because of the production and elaboration of toxic secondary metabolites. The genus *Aspergillus* comprising of *A. flavus*, *Aspergillus* section *Nigri*, *A*. *oryzae*, *A*. *parasiticus*, *A. terreus*, and *A*. *versicolor* are frequent contaminants in agricultural commodities like paddy, milled rice, peanuts, maize, millets, and wheat. These *Aspergilli* produce several mycotoxins at different relative humidities and storage periods, namely aflatoxin B_1_, B_2_, G_1_, G_2_, ochratoxin A, and fumonisin B_1_ (Amadi and Adeniyi, 2009). Aflatoxins are toxigenic, carcinogenic, mutagenic, teratogenic, immunosuppressive, and are produced by *Aspergilli* as by-products which can also contaminate crops like peanut and cotton (Bhatnagar-Mathur et al., 2015; Soni et al., 2020) in addition to spices. Of the multitude of mycotoxins produced by *Aspergillus* species, aflatoxins B_1_ (Nurtjahja et al., 2019), fumonisin B_2_ (Han et al., 2017), and ochratoxin A (Magnoli et al., 2007), seem to be quite stable during storage of seeds and spices. Despite extensive toxicological studies with aflatoxins and other mycotoxins, the significance to human health, in case of several mycotoxins remains unclear. Synergistic interactions due to the co-occurrence of toxins is yet to be established.

*Aspergillus* species have medical and commercial importance. The genus *Aspergillus* has economic importance in different aspects like fermentation industry and enzyme production (Malathi and Chakraborty, 1991). Members of the genus produce a wide range of secondary metabolites, including lovastatin, a cholesterol-lowering drug, antitumor metabolites, etc. (Casas López et al., 2004). Few members of the genus are also the source of natural products which can be used to treat human diseases (Hiort et al., 2004).

### Challenges in Identification of *Aspergillus* Species by Morphological Characterization

Mycologists have traditionally used phenotypic characteristics as a sole means for fungal identification. Classification of *Aspergilli*, which comprise highly speciose lineages, requires techniques such as fungal barcoding for accurate species identification. *Aspergilli* have been recognized since ancient times by the formation of a common morphological structure referred to as the “*Aspergillum*” which consists of an asexual reproductive and distinctive entity, with a characteristic stalk-like “conidiophore” bearing a bulbous vesicle, which in turn bears the phialides and metulae, on which chains of microconidia are arranged (Bennett, 2010) in basipetal succession. Morphological identification of *Aspergillus* spp. by using macro-morphological characters like colony diameter, color, obverse, and reverse colony characters, etc., and microscopic features like conidial heads, vesicles shape, length of vesicles, stipes, seriation, metula, conidia color, size shape and ornamentations, asci, ascospore size, shape, color, etc., would only help in the preliminary assignment of generic names, leading to an inadequacy in identification to assign species. The limitations posed by morphological identification are many. These tests are extremely time consuming, cumbersome, requiring skilled personnel and expertise in carrying out the whole process. Hence, fungal phylogeny which uses genetic and molecular tools, are being used alongside the conventional techniques to address the gap of misidentification (Raja et al., 2017).

To overcome this constraint, a set of adoptable standard procedures like the use of nuclear ribosomal genes (ITS) most commonly used in fungal identification, considered as the official barcoding marker and methods used for construction of phylogenetic tree, which facilitates species identification has been carried out (Raja et al., 2017). The ITS and beta-tubulin genes have not been systematically used in *Aspergillus* taxonomy from *F*. *vulgare* till date, and have thus been appropriately evaluated in this study. In addition, our purpose was to examine protein-coding functional sequences as a major genetic marker for assessing intraspecies diversity. Therefore, the beta-tubulin gene is being considered in the current study since researchers (Cleveland and Sullivan, 1985) have reported that multiple tubulin genes are required in all but the simplest eukaryotic organisms (Edgcomb et al., 2001), for the formation, maintenance and preservation of the essential microtubules. The beta-tubulin gene appears to occur as a single-copy (α_1_, α_2_, β_1_, γ tubulins) in *Aspergillus niger*, single-copy (α_1_, γ tubulins), two-copy (β_1_) or three-copy sequences (α_2_ tubulins) in *Aspergillus nidulans* (Zhao et al., 2014). Among the various genes with basic functionality of coding for proteins in all eukaryotes (Mages et al., 1995; Schütze et al., 1999), the beta-tubulin gene (Keeling et al., 2000; Ayliffe et al., 2001; Mukherjee et al., 2003; Juuti et al., 2005), is also essential for sexual development in *A*. *nidulans* (Kirk and Morris, 1991) and has received enormous attention due to its highly conserved nature. This is especially important while phylogenetic constructions are undertaken with a broad range of organisms or for a specific genus.

Single nucleotide polymorphisms (SNPs) are single-nucleotide substitutions of one base for another and are definitely the most prevalent set of genetic variety. There are two sorts of techniques to examine SNPs: genomic and functional. SNPs have a variety of consequences, for instance, drug resistance, effects on mRNA splicing, nucleocytoplasmic export, etc. There is no information available on SNPs in *Aspergilli* isolated from spices. The current analysis has made efforts to identify synonymous and non-synonymous mutations, to examine them evolutionarily, and to reclassify *Aspergilli* into their respective Sections for appropriate taxonomic identification.

With this background, the predominant objectives of this study constituted, the elaborate and detailed macro- and micro-morphological studies of *Aspergillu*s species, molecular characterization of *Aspergillus* isolates obtained from *Foeniculum vulgare* samples using universal fungal bar-coding of the Internal Transcribed Spacer (ITS) and a functional genetic marker namely, beta–tubulin (β) gene sequences. This analysis was also performed with the primary objective of molecular fungal barcoding to substantiate our morphological analysis. Further, phylogenetic and single nucleotide polymorphism analyses have also been performed, for the first time from *Aspergilli* isolated from Indian fennel seed samples.

## Materials and Methods

### Collection of Samples

A total number of seventy (70) samples of the Indian spice *Foeniculum vulgare* were collected from different retail markets from several regions, namely New Delhi, Lucknow, Pinjore, West Midnapore, and Puducherry from India. These samples were from urban, semi-urban and rural zones from different geographical regions of India. The samples were purchased in portions of 100 g and stored in sterile polyethylene zip-lock pouches. All samples were adequately labeled with suitable codes. The pouches were then transported to the laboratory and analyzed sequentially at the earliest. Representative spice samples were stored at 4°C for further analysis.

### Macro-Morphological Cultural Studies of *Aspergilli*

Standard methods of agar plating technique as described by the International Seed Testing Association (ISTA) were chosen for the mycofloral analyses of the Indian fennel samples. Briefly, 25 g of the spice samples was weighed, washed thrice with distilled water and then rinsed with 1% sodium hypochlorite (NaOCl) solution for one min to inactivate surface contaminants. Subsequently, the seeds were rinsed in distilled water to remove any adhering sodium hypochlorite. The seeds were briefly allowed to stand for about 5–10 min after they had been placed in sterile petriplates lined with sterile tissue paper and plated using standard procedures. The petri plates containing the seeds were incubated in upright position at 25–27°C for 7 days. The results were expressed as percentage of infection and the frequency of occurrence was calculated (Reddy et al., 2009; dos Santos-Ciscon et al., 2019).

*Aspergillus* isolates were obtained after plating *Foeniculum vulgare* spice samples on mycological/fungi-specific media namely fungal agar (FA) and potato dextrose agar (PDA) media (HiMedia, Mumbai, India) by following standard agar plating procedures (Xie et al., 2007; Hamzah et al., 2018). All media were prepared with the mandatory addition of a thermostable, antibacterial antibiotic namely chloramphenicol (Sigma-Aldrich, Steinhein, Germany) at the rate of 20 mg/1,000 mL of the fungi-specific media proposed. Briefly, representative samples were washed thrice with distilled water, dried for about 15 min and then plated @ 25 seeds per petri plate (100 × 15 mm, S-line, Borosil^®^, Mumbai, India), using aseptic techniques. The plates were then incubated at 25°C ± 2°C for 5–7 days with alternating periods of 12 h light and 12 h darkness. On the 7th day, *Aspergillus* colonies (green, greenish yellow, ochre/yellow, black, brownish-black, dark-brown, biscuit-brown, blue) were observed and isolated from the spice samples onto PDA medium. Spore suspensions of the *Aspergilli* were prepared and about 20 μL was transferred onto fresh PDA media. Colonies arising from single germinating spores were checked using Stereo Binocular Microscope (Magnus MSZ-Bi, Model: 13M1009), chosen for further studies and identified based on descriptions made in Fungal keys and Manuals (Thom and Raper, 1945; Varga and Samson, 2008) and Monographs (Refai et al., 2014).

### Macro-Morphological Microscopic Studies of *Aspergilli*

Stereo binocular microscopic observations were carried out to detect presence of different species of *Aspergilli* and distinguish them from other fungal genera like *Alternaria*, *Curvularia*, *Fusaria*, *Penicillia*, *Mucor, Mycelia sterilia*, *Trichothecium*, etc., encountered during the analysis. Fungal flora were isolated from the fennel samples on potato dextrose agar (PDA) and czapek dox agar (CDA) media and identified using fungal manuals and keys (Thom and Raper, 1945; Refai et al., 2014). While mycoflora were being studied, emphasis was laid to the observation and isolation of *Aspergilli*. Macromorphological studies were carried out on aforesaid agar media. The isolates were inoculated either by single point or three-point inoculation method in glass petriplates (100 × 15 mm, S-line, Borosil^®^, Mumbai, India). After 7 days of incubation colony diameters, color, texture, sporulation pattern, obverse and reverse colony colors/characters and presence or absence ascomata (in older cultures incubated for 15–25 days) were determined.

### Micro-Morphological Microscopic Studies of *Aspergilli*

The microscopic features of the isolated *Aspergilli* were sequentially studied using Light Microscopy (Olympus CH20i), Scanning Electron (SE; Hitachi, Model E-1010) and Differential Interference Contrast (DIC) microscopic analysis. Prominence was given to study characteristics like hyphal nature, septa, conidiophores, vesicle shape, sterigmata arrangement, and conidia formation. Standard protocols were followed while studying the microscopic features as outlined in identification Keys and Manuals (Thom and Raper, 1945; Varga and Samson, 2008). The light and scanning electron micrographs were taken at the Fungal Genetics and Mycotoxicology Laboratory, Department of Microbiology, Pondicherry University, while the DIC microscopic images (Nikon Upright Motorised Microscope, ECLIPSE Ni series, Nikon Corporation, Tokyo) were taken at the Fungal Biotechnology Laboratory, Department of Biotechnology, School of Life Sciences, Pondicherry University.

### Preparation of Fungal Cultures for Genomic DNA Isolation

As and when microscopic analyses were being performed pure cultures of *Aspergilli* were inoculated onto PDA medium and incubated at 28°C for 7 days. Fungal mycelium from each petriplate was scraped off the medium using disposable sterile blades (Carbon steel Scalpel Blade No. 11, Olrada, India) and transferred to sterile pestle and mortar. The mycelial biomass (approximately close to 100 mg was collected) was used for DNA isolation.

### Isolation of Fungal Genomic DNA

DNA was isolated from fungal isolates grown on synthetic PDA plates and DNA isolation was carried out using a Standard Kit (Gene JET plant Genomic DNA Purification Kit- K0791, Thermo Fisher Scientific, Vilnius, Lithuania). 100 mg of the fungal mycelial biomass was transferred to a sterile pestle and mortar. To this liquid nitrogen (200 mL) was carefully added along the walls of the mortar and crushed adequately in clockwise fashion for 5 min. The crushed mycelial biomass was immediately transferred to a new, sterile microcentrifuge tube (1.5 mL; Tarsons, Kolkata, India). Soon after, Lysis Buffer A (350 μL) and Lysis Buffer B (50 μL) were added in succession. A tiny quantity of RNase A (20 μL) was transferred to the microcentrifuge tubes containing the lysis buffers and crushed mycelial biomass. The specimen was then subjected to heat treatment for 10 min at 65°C using a sterile water bath with occasional stirring. After the heat treatment, the precipitation solution (130 μL) was added as per manufacturer’s instructions. The microcentrifuge tubes were adequately mixed and kept on frost for 5 min. The tubes were then spun at ≥ 20,000 g (≥ 14,000 rpm) for 5 min. The tubes were removed, the supernatant (precisely 450–550 μL) was collected and transferred to a clean microcentrifuge tube, and an equal volume of plant gDNA binding solution and 96% ethanol (400 μL) was added and vigorously mixed. Thereafter, 600–700 μL of the prepared solution was transferred, isolate-wise, to the new spin columns. The supernatant thus collected was centrifuged at 6,000 g (∼8,000 rpm) for about a min. Using the same column, the supernatant solution was removed and the remaining mixture was centrifuged for 1 min. The wash buffer I (500 μL) was added into the spin-columns and centrifuged at 8,000 g (∼10,000 rpm) for 1 min, followed by the addition of wash buffer II (500 μL) in the spin columns again. This time the spin columns were centrifuged at high speed of ≥ 20,000 g (≥ 14,000 rpm) for 3 min. The contents of the collection tube within the spin columns were discarded, and the column contents were transferred to fresh, new microcentrifuge tubes. For genomic DNA elution, the elution buffer (30 μL) was placed mid-point of the spin column layer, incubated at room temperature for 5 min. The microcentrifuge tubes were spun at a speed of 8,000 g (∼10,000 rpm) for 1 min. A second elution step was carried out using the elution buffer (20 μL). The purified DNA was stored at −20°C for further use.

### Polymerase Chain Reaction of the Internal Transcribed Spacer and β-Tubulin Genes

A total number of 27 isolates were selected for a phylogenetic analysis of the ITS and β-tubulin gene using (Tam et al., 2014) as one of the references. Partial amplification of the Internal Transcribed Spacer (ITS) region and functional gene β-tubulin was performed using the primers ITS1 (5′-TCCGTAGGTGAACCTGCGG-3′) and ITS4 (5′-TCCTCCGCTTATTGATATGC-3′) for ITS (Op De Beeck et al., 2014; Tam et al., 2014), bT2a (5′- GGTAACCAAATCGGTGCTGCTTTC-3′) and bT2b (5′- ACCCTCAGTGTAGTGACCCTTGGC-3′) for β-tubulin (Tam et al., 2014) respectively. Polymerase Chain Reaction (PCR) reactions were performed in a 25 μL volume, containing 12.5 μL (Taq DNA Polymerase 2x Master Mix RED, Ampliqon PCR Enzymes and Reagents, Stenhuggervej, Denmark), Forward primer (10 pmoL) 2.0 μL, Reverse primer (10 pmoL) 2.0 μL, Template DNA X μL (genomic DNA: 10–500 ng), Molecular biology grade water X μL (HiMedia^®^, Mumbai, India) for ITS gene, 12.5 μL (Taq DNA Polymerase 2x Master Mix RED, Ampliqon PCR Enzymes and Reagents), Forward primer (10 μM) 1.0 μL, Reverse primer (10 μM) 1.0 μL, Template DNA X μL (genomic DNA: 10–500 ng), Molecular biology grade water X μL (HiMedia^®^, Mumbai, India) for β-tubulin gene amplification. The cycling protocol consisted of an initial denaturation step of 94°C for 1 min 30 s, 32 cycles of denaturation at 95°C for 35 s, annealing for 55 s at 55°C for ITS and an initial denaturation step of 95°C for 3 min, 32 cycles of denaturation at 94°C for 30 s, annealing for 45 s at 65.5°C for β-tubulin and extension at 72°C for 1 min followed by a final extension of 10 min for ITS and 13 min for β-tubulin at 72°C. PCR products were sent to (Biokart India Pvt. Ltd., Bangalore, India) for purification and sequencing.

### Phylogenetic Analyses

A consensus sequence for each locus of ITS and partial β-tubulin gene was generated using the forward and reverse sequence with CAP3 programme^1^ (Huang and Madan, 1999). Newly generated sequences have been deposited in GenBank^2^ (Table 1). A similarity search was performed for the newly generated sequences using BLASTn of the National Centre for Biotechnology Information.^3^ The homologous sequences for each isolate belonging to the corresponding species were retrieved from the GenBank database. The retrieved sequences were aligned using MAFTT v 7^4^ (Katoh and Standley, 2013) with default parameters and alignments were edited with BioEdit v7.2.5.0 (Hall, 1999) and were manually adjusted as and when required. The low-quality bases at the start and end positions of the sequences were removed.

**TABLE 1:** Section-wise details of *Aspergillus* isolates used in the current study, GenBank accession numbers of ITS and β-tubulin gene sequences.

Sl. No.	FGM lab isolate code	Section	Anamorph/Teleomorph	Accession number (ITS)	Accession number (β-tubulin)
1	51	*Terrei*	*Aspergillus aureoterreus*	−	MN791093
2	18		*Aspergillus aureoterreus* *	−	MN791096
3	1		*Aspergillus aureoterreus*	−	MN791109
4	16		*Aspergillus terreus* *	MN392907	MN791095
5	52		*Aspergillus terreus*	MN264636	−
6	61	*Flavi*	*Aspergillus flavus* *	−	MN791106
7	22		*Aspergillus tamarii* *	MN326529	MN791098
8	75		*Aspergillus tamarii*	MN317364	MN791108
9	S44		*Aspergillus tamarii*	−	MN791115
10	63		*Aspergillus tamarii*	MN263247	−
11	24	*Fumigati*	*Aspergillus fumigatus*	−	MN791099
12	37		*Aspergillus fumigatus*	MN317367	MN791100
13	31		*Aspergillus fumigatus* *	MN264637	MN791103
14	17	*Nidulantes*	*Aspergillus nidulans*	−	MN791097
15	45		*Aspergillus nidulans* *	MN309877	MN791101
16	9		*Aspergillus nidulans*	−	MN791102
17	71		*Aspergillus nidulans*	MN317365	MN791107
18	2		*Aspergillus nidulans/Emericella nidulans*	−	MN791116
19	49		*Aspergillus quadrilineatus/Emericella quadrilineata*	−	MN791104
20	58		Aspergillus quadrilineatus*/Emericella quadrilineata	−	MN791105
21	4		*Aspergillus latus* *	−	MN791110
22	11		*Aspergillus latus*	−	MN791111
23	12		*Aspergillus latus*	−	MN791112
24	S8	*Nigri*	*Aspergillus awamori*	−	MN791113
25	S24		*Aspergillus awamori* *	−	MN791114
26	83	*Versicolores*	*Aspergillus sydowii* *	MN298848	−
27	29	Unassigned	*Aspergillus* species	MN294688	−

*Isolates subjected to macro- and micro-morphological analyses.

*ITS, Internal Transcribed Spacer gene; FGM Laboratory, Fungal Genetics and Mycotoxicology Laboratory; All fungal isolates were isolated, cultured and studied by Pranab Kumar Mahata and Regina Sharmila Dass.*

The retrieved homologous sequences for each genetic marker were aligned separately with ClustalX2 v2.1 (Thompson et al., 1997) using default parameters. Phylogenetic analyses of individual loci were performed by maximum parsimony (MP) method using PAUP v 4.0 beta win (Swofford and Sullivan, 2003) (heuristic search option with 1,000 random taxon additions and tree bisection and reconnection (TBR) as the branch swapping algorithm). The efficacy and the robustness of the parsimonious trees were evaluated with 1,000 bootstrap replications (Hillis and Bull, 1993). Further, the maximum likelihood (ML) method was used for the analysis of individual loci via the CIPRES Science Gateway.^5^ Analysis was carried out using RA_x_ML v. 7.4.2 (Stamatakis et al., 2012). A general time-reversible model (GTR) was applied, including estimation of invariable sites and a discrete gamma distribution with four rate classes (GTRG + I). The reproducibility of the branches was evaluated by bootstrap analysis with 1,000 replicates.

The trees that were being generated were envisaged with FigTree v1.4.0 (Rambaut and Drummond, 2009). Bootstrap (bs) percentages of analysis were labeled at the nodes. Values less than 70% bs were removed. Branches with values 95% and above bs were thickened.

### Single Nucleotide Polymorphisms

Each of the sequences of the fungal isolates listed in Table 1 were aligned with the reference genomes of respective species that are available on the NCBI using BLAST algorithm. Variants observed in these sequences were fetched manually and the corresponding variants on the protein sequences were annotated manually from the protein annotations of β-tubulin genes of the respective species.

### Statistical Analysis

To learn more about the *Aspergillus* fungal isolates, we employed descriptive statistics on the data. The statistical studies were carried out using Origin 8.6 (Northampton, United States).

## Results

### Relative Abundance in *Foeniculum vulgare*

**FIGURE 1:**
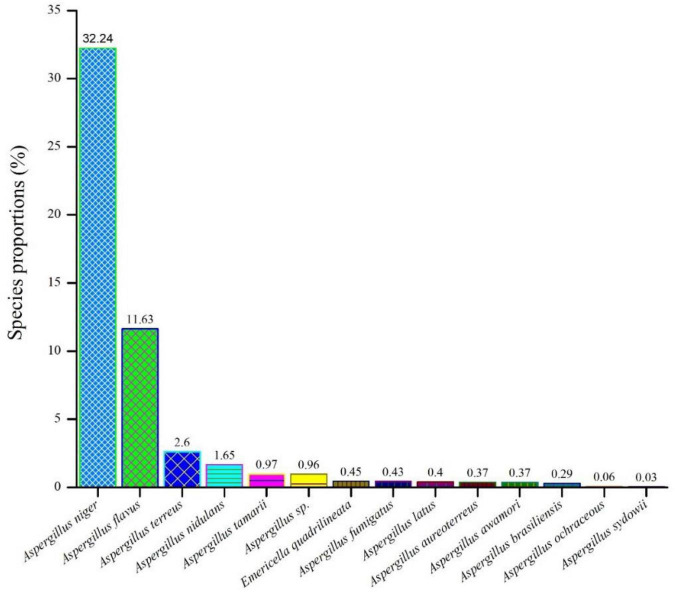
An assessment of the relative abundances of several *Aspergillus* species in the *F*. *vulgare* samples.

As shown in Figure 1 among all the isolates collected from *F. vulgare* (n = 70), we were able to identify fourteen (14) different species of the *Aspergillus*. *Aspergillus niger* (32.24%) ranked first, followed by *A. flavus* (11.63%), *A. terreus* (2.6%), *A. nidulans* (1.65%), *A. tamarii* (0.97%), *A. species* (0.96%), *Emericella quadrilineata* (0.45%), *A. fumigatus* (0.43%), *A. latus* (0.4%), *A. aureoterreus* (0.37%), *A. awamori* (0.37%), *A. brasiliensis* (0.29%), *A. ochraceous* (0.06%), and *A. sydowii* (0.03%).

### *Aspergillus* Diversity

**FIGURE 2:**
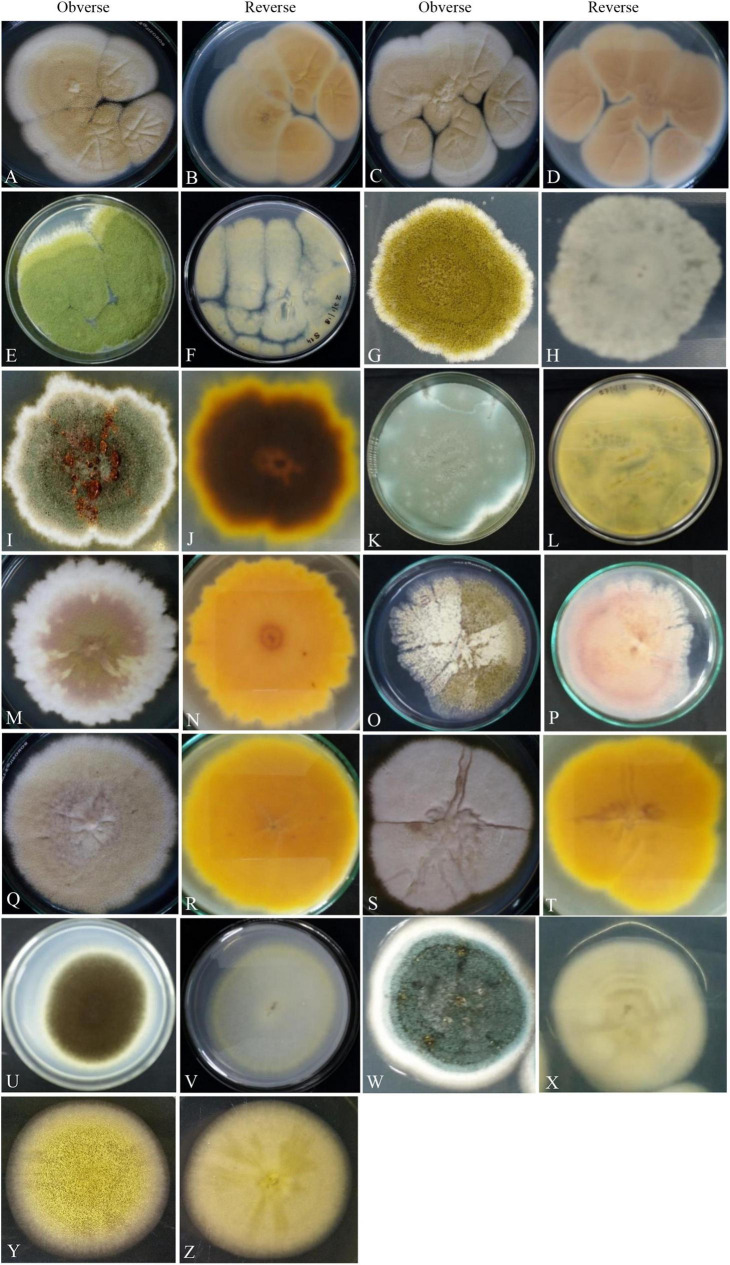
Photographs illustrating the macromorphology of strains of *Aspergillus aureoterrus* **(A,B)** (Acc. No. MN791096); *Aspergillus terreus* **(C,D)** (Acc. No. MN791095); *Aspergillus flavus* **(E,F)** (Acc. No. MN791106); *Aspergillus tamarii* **(G,H)** (Acc. No. MN791098); *Aspergillus fumigatus* **(I,J)** (Acc. No. MN791103); *Aspergillus fumigatus* **(K,L)** (Acc. No. MN791100); *Aspergillus nidulans* **(M–P)** (Acc. No. MN791101); *Aspergillus quadrilineatus/Emericella quadrilineata* **(Q,R)** (Acc. No. MN791105); *Aspergillus latus* **(S,T)** (Acc. No. MN791110); *Aspergillus awamori* **(U,V)** (Acc. No. MN791114); *Aspergillus sydowii* **(W,X)** (Acc. No. MN298848), and *Aspergillus* species **(Y,Z)** (Acc. No. MN294688) are shown. Images **(O,P)** of *Aspergillus nidulans* were incubated in CDA for 7 days, whereas all other isolates were kept in PDA.

**FIGURE 3:**
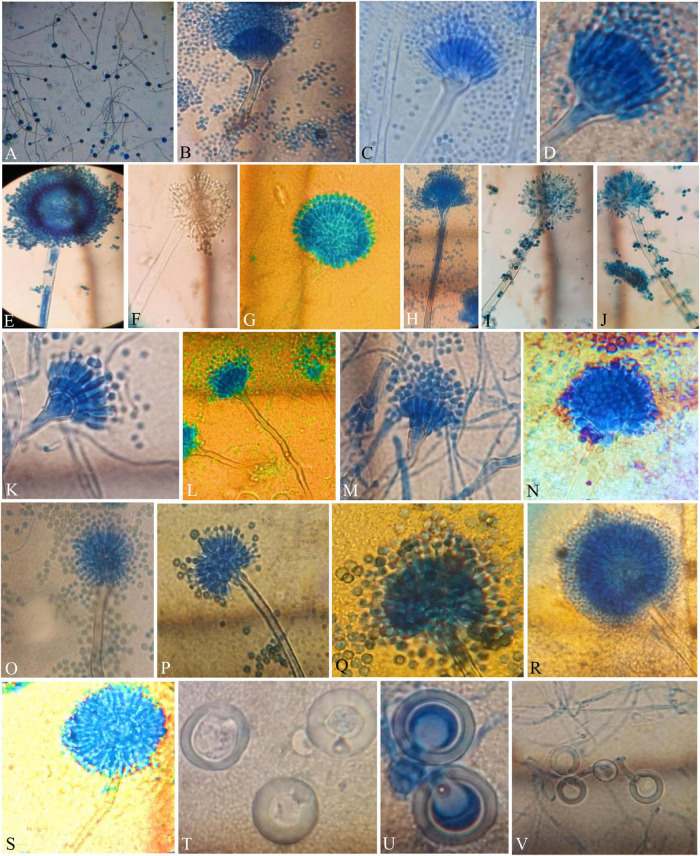
Compound Light Microscope (CLM) images of the hyphae, conidiophores, and Hülle cells, **(A,B)** hyphae and elongated conidiophores of *A. aureoterreus*; **(C,D)** enlarged conidiophores of *A. terreus*; **(E,F)** stained and unstained conidiophores of *A. flavus*; **(G)** enlarged conidial head of *A. tamarii*; **(H–J)** conidiophores with bottle-shaped vesicles, uniseriate sterigmata, and conidia of *A. fumigatus*; **(K)** enlarged conidiophores of *A. nidulans*; **(L)** conidiophores of *E. quadrilineata*; **(M)** conidiophores of *A. latus*; **(N,O)** *A. awamori* conidiophores; **(P,Q)** *A. sydowii* conidiophores; **(R,S)** *Aspergillus* species conidiophores, and **(T–V)** *A. nidulans*, *E. quadrilineata*, and *A. latus* Hülle cells, respectively.

**FIGURE 4:**
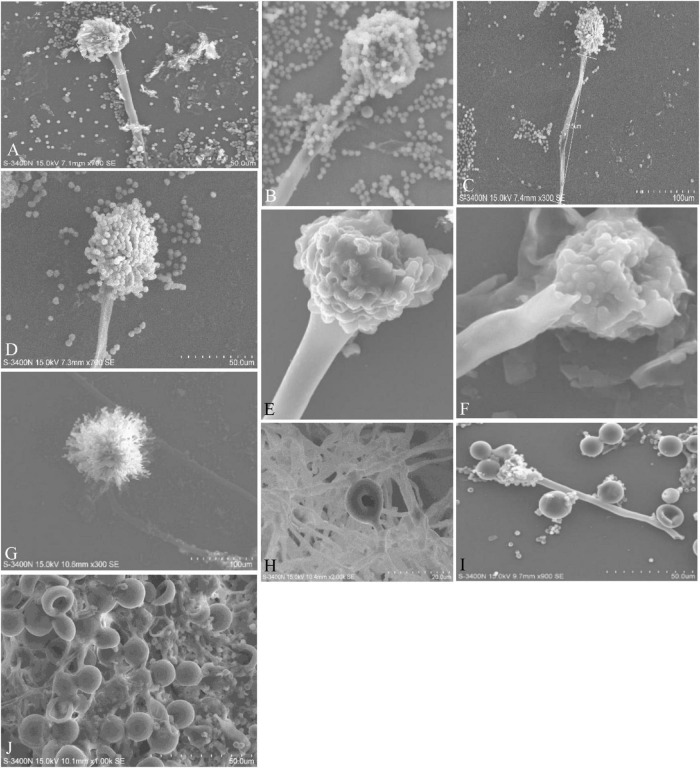
Conidiophores, vesicles, metulae, phialides, conidia, and Hülle cells of the strains are shown in Scanning Electron Microscope (SEM) images. **(A)** Conidiophores of *A. aureoterreus*; **(B)** conidiophores of *A. terreus*; **(C,D)** conidial head with chains of conidia of *A. tamarii*; **(E)** conidiophores of *A. nidulans*; **(F)** conidiophores of *A. latus*; **(G)** globose conidial heads, with metulae, phialides, chains of conidia and smooth conidiophores in *A. awamori*; **(H,I)** mycelium and Hülle cell development and Hülle cells of *A. nidulans* **(J)** Hülle cells formation in *E. quadrilineata.*

**FIGURE 5:**
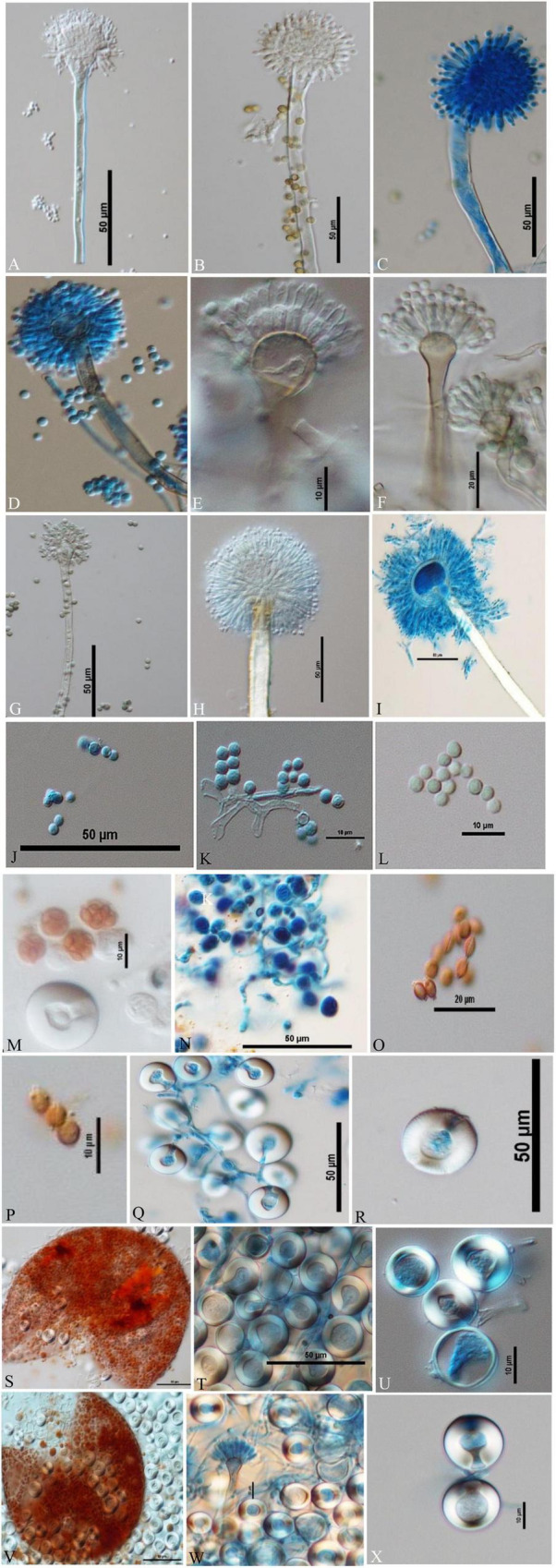
Conidiophores, conidia, asci, ascospores, cleistothecia, and H lle cells Differential Interference Contrast (DIC) images of *Aspergilli* **(A)** *A. aureoterreus* smooth, long, colourless conidiophores and columnar, biseriate conidial heads with hemispherical vesicles, metulae phialides, and conidia; **(B,C)** *A. tamarii* conidiophores containing globose, radiating and rough conidia, globose vesicles with uniseriate sterigmata; **(D)** *A. nidulans* smooth-walled, sinuate conidiophores and columnar conidial heads with small hemispherical vesicles, metulae, phialides, and conidia; **(E)** *E. quadrilineata* smooth, sinuate conidiophores and short, columnar conidial heads with hemispherical vesicles, metulae, phialides, and conidia; **(F)** *A. latus* smooth-walled, sinuate conidiophores and columnar conidial heads with small hemispherical vesicles, metulae, phialides, and conidia; **(G)** *A. sydowii* smooth, sinuous conidiophores and hemispherical conidial heads with globose to elliptical vesicles, metulae, phialides, and conidia; **(H,I)** *Aspergillus* species conidiophores with rough, pitted surfaces, globose conidial heads with globose, thinner vesicles, metulae, phialides, and conidia; **(J–L)** globose conidia of *A. nidulans*, *E. quadrilineata*, and *A. latus*, respectively; **(M,N)** asci of *A. nidulans* and *A. latus*; **(O)** lenticular ascospores of *A. latus*; **(P)** lenticular, smooth walled (unstained) ascospores of *E. quadrilineata*; **(Q,R)** Hülle cells of *A. nidulans*; **(S–U)** ruptured cleistothecium and Hülle cells of *E. quadrilineata*, and **(V)** in *A. latus*, ruptured cleistothecium showing asci, surrounded by Hülle cells, **(W,X)** Hülle cells separated from *A. latus*.

The cultivation of fungi from seventy (70) fennel samples yielded a total of 3,506 *Aspergillus* isolates. Apart from *Aspergilli*, genera like *Alternaria* species, *Curvularia* species, *Drechslera* species, *Fusarium* species, *Mucor* species, *Mycelia sterilia*, *Neurospora crassa*, *Penicillium* species, *Trichothecium* species and Yeasts were also isolated based purely on morphological and microscopical analyses. The detailed cultural morphology and microscopic analyses of the *Aspergilli* has been carried out and has been presented with descriptions (Figures 2–5). *A*. *niger* (Relative abundance: 32.24%) followed by *A*. *flavus* (Relative abundance: 11.63%) were found to be the two most frequently occurring species. The fourteen (14) *Aspergillus* species were isolated from Indian fennel seeds, collected from different geographical zones of India, and studied in detail using morphological and genetic methods for molecular phylogeny. The Internal Transcribed Spacer gene of the ribosomal DNA (rDNA-ITS) sequencing identified 20 isolates under five *Aspergillus* Sections namely *Terrei*, *Versicolores*, *Flavi*, *Fumigati* and *Nidulantes* (Sub-genus II) and corresponding to the family *Trichocomaceae*. The isolates included *A*. *nidulans*, *A*. *quadrilineatus*, *A*. *miyajii*, *A*. *rugulosus*, *A*. *terreus*, *A*. *tamarii*, *A*. *fumigatus*, *A*. *sydowii* and *Aspergillus* species (Section Unassigned). Isolates sequenced for β-tubulin gene sequences revealed the identity of 23 *Aspergilli* with species namely *A*. *nidulans*, *A*. *quadrilineatus*, *A*. *latus*, *A*. *terreus*, *A*. *aureoterreus*, *A*. *flavus*, *A*. *tamarii*, *A*. *fumigatus* and *A*. *awamori* from five sections namely *Nidulantes*, *Terrei*, *Flavi*, *Fumigati*, and *Nigri* (Sub-genus I) with no known teleomorphs. The *Aspergilli* namely *A*. *quadrilineatus*, *A*. *latus*, *A*. *aureoterreus*, *A*. *awamori* and *Aspergillus* species are being reported for the very first time from fennel seeds. The details of the GenBank depositions are also shown (Table 1).

### Phylogenetic Analyses

**FIGURE 6:**
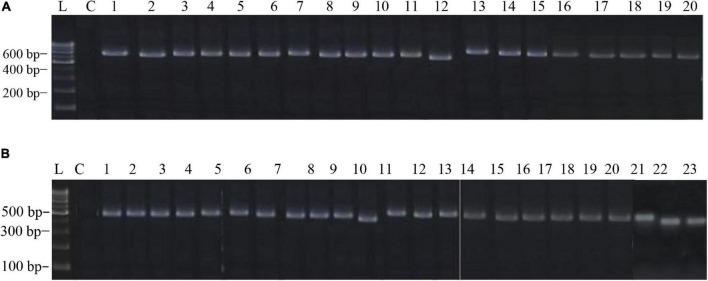
**(A)** The electrophoretogram shows the positive PCR amplification of *Aspergillus* ITS genes with species-specific amplicons. Here, L, 100-bp MBT049 HIMEDIA DNA ladder; C, PCR with a negative control; 1, 49-*Aspergillus nidulans*; 2, 58-*Aspergillus nidulans*; 3, 63-*Aspergillus tamarii*; 4, 31-*Aspergillus fumigatus*; 5, 37-*Aspergillus fumigatus*; 6, 45-*Aspergillus nidulans*; 7, 51-*Aspergillus terreus*; 8, 52-*Aspergillus terreus*; 9, 71-*Aspergillus nidulans*; 10, 75-*Aspergillus tamarii*; 11, 83-*Aspergillus sydowii*; 12, 17-*Aspergillus miyajii*; 13, 16-*Aspergillus terreus*; 14, 22-*Aspergillus tamarii*; 15, 29-*Aspergillus sp.;* 16, 4-*Aspergillus quadrilineatus*; 17, 2-*Aspergillus rugulosus*; 18, 1-*Aspergillus terreus*; 19, 11-*Aspergillus nidulans*; 20, 12-*Aspergillus nidulans*. **(B)** The electrophoretogram shows the positive PCR amplification of *Aspergillus* β-tubulin genes with species-specific amplicons. Here, L, 100-bp MBT049 HIMEDIA DNA ladder; C, PCR with a negative control; 1, 49-*Emericella quadrilineata*; 2, 58-*Emericella quadrilineata*; 3, 61-*Aspergillus flavus*; 4, 31-*Aspergillus fumigatus*; 5, 37-*Aspergillus fumigatus*; 6, 45-*Aspergillus nidulans*; 7, 51-*Aspergillus aureoterreus*; 8, 71-*Aspergillus nidulans*; 9, 75-*Aspergillus tamarii*; 10, 18-*Aspergillus aureoterreus*; 11, 17-*Aspergillus nidulans*; 12, 16-*Aspergillus terreus*; 13, 9-*Aspergillus nidulans*; 14, 24-*Aspergillus fumigatus*; 15, 22- *Aspergillus tamarii;* 16, 4-*Aspergillus latus*; 17, 2-*Emericella nidulans*; 18, 1-*Aspergillus aureoterreus*; 19, 11- *Aspergillus latus*; 20, 12-*Aspergillus latus*; 21, S8-*Aspergillus awamori*; 22, S24-*Aspergillus awamori*; 23, S44-*Aspergillus tamarii*.

**FIGURE 7:**
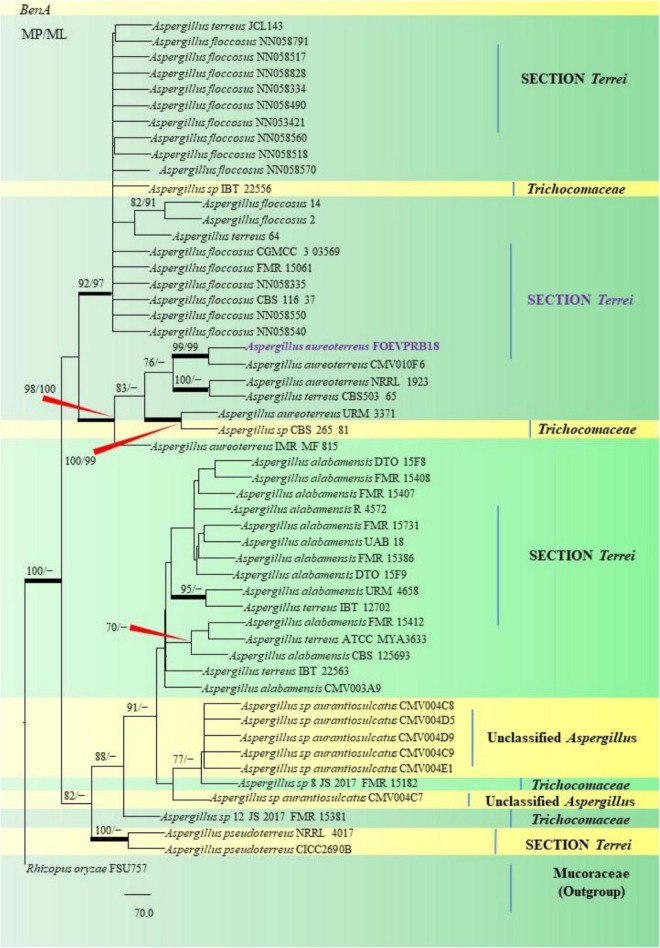
A phylogenetic tree (taxon branching diagram) presents the genetic analysis of the *Aspergillus aureoterreus* FOEVPRB18 clade belonging to the Section *Terrei*, exhibiting a maximum parsimony analysis of partial β-tubulin concatenated sequences, acquired. Bootstrap (bs) values ≥ 70% are designated at the nodes for maximum parsimony (MP) and maximum likelihood (ML) methods; branches with values ≥ 95% bs are bold black. A specific isolate code was mentioned as a suffix to the species name. Hyphen (-) represents the range of support values for parsimony and RAxML analyses of below 70%. Our isolate, which is marked in purple, was rooted using *Rhizopus oryzae* FSU757.

**FIGURE 8:**
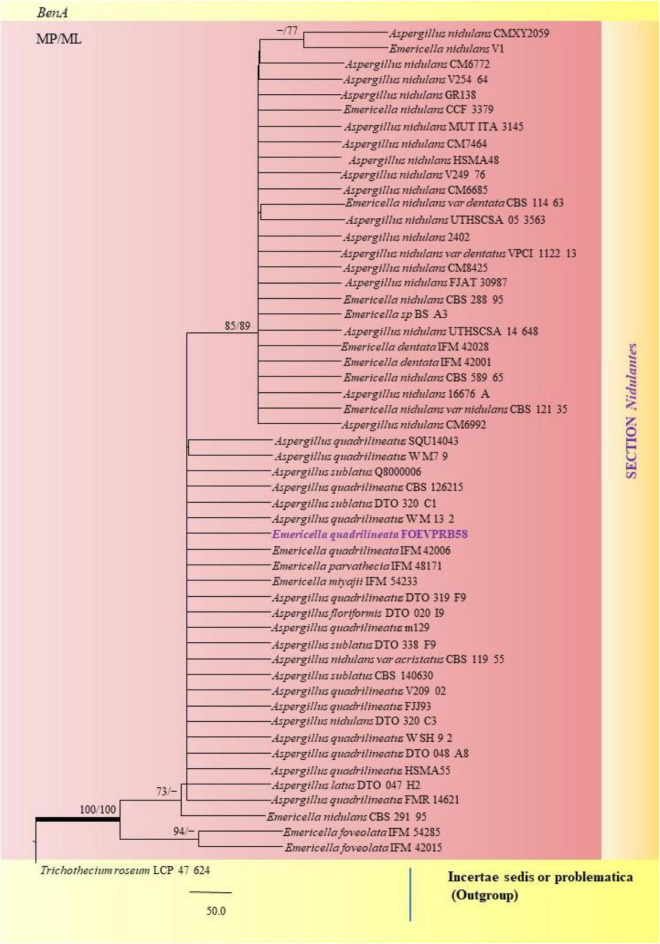
A phylogenetic tree (taxon branching diagram) which presents the genetic analysis of the *Emericella quadrilineata* FOEVPRB58 clade belonging to the Section *Nidulantes*, exhibiting a maximum parsimony analysis of partial β-tubulin concatenated sequences, acquired. Bootstrap (bs) values ≥ 70% are designated at the nodes for maximum parsimony (MP) and maximum likelihood (ML) methods; branches with values ≥ 95% bs are bold black. A specific isolate code was mentioned as a suffix to the species name. Hyphen (-) represents the range of support values for parsimony and RAxML analyses of below 70%. Our isolate, which is marked in purple, was rooted using *Trichothecium roseum* LCP 47 624.

**FIGURE 9:**
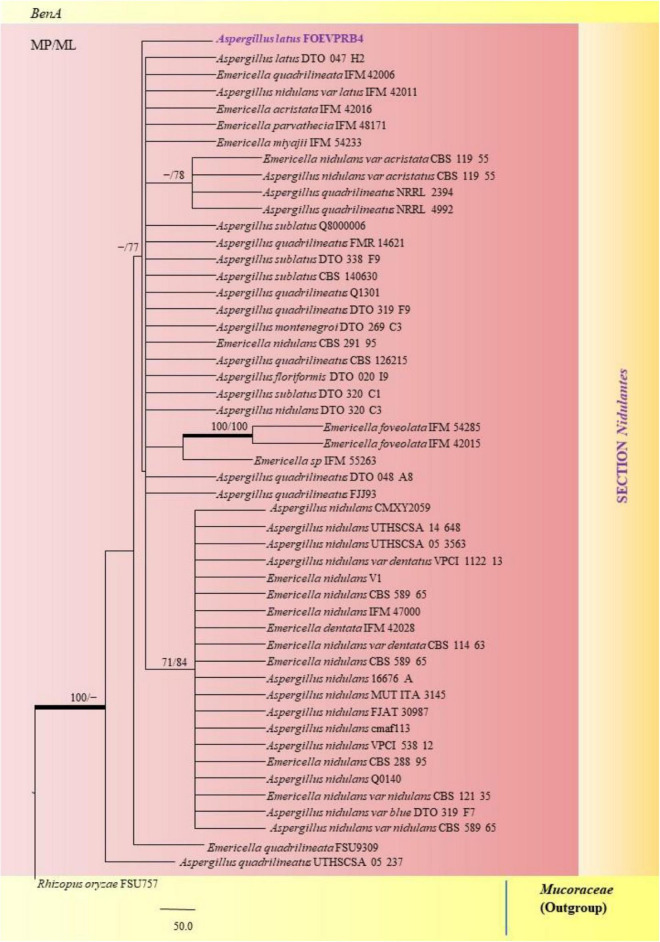
A phylogenetic tree (taxon branching diagram) which presents the genetic analysis of the *Aspergillus latus* FOEVPRB4 clade belonging to the Section *Nidulantes*, exhibiting a maximum parsimony analysis of partial β-tubulin concatenated sequences, acquired. Bootstrap (bs) values ≥ 70% are designated at the nodes for maximum parsimony (MP) and maximum likelihood (ML) methods; branches with values ≥ 95% bs are bold black. A specific isolate code was mentioned as a suffix to the species name. Hyphen (-) represents the range of support values for parsimony and RAxML analyses of below 70%. Our isolate, which is marked in purple, was rooted using *Rhizopus oryzae* FSU757.

**FIGURE 10:**
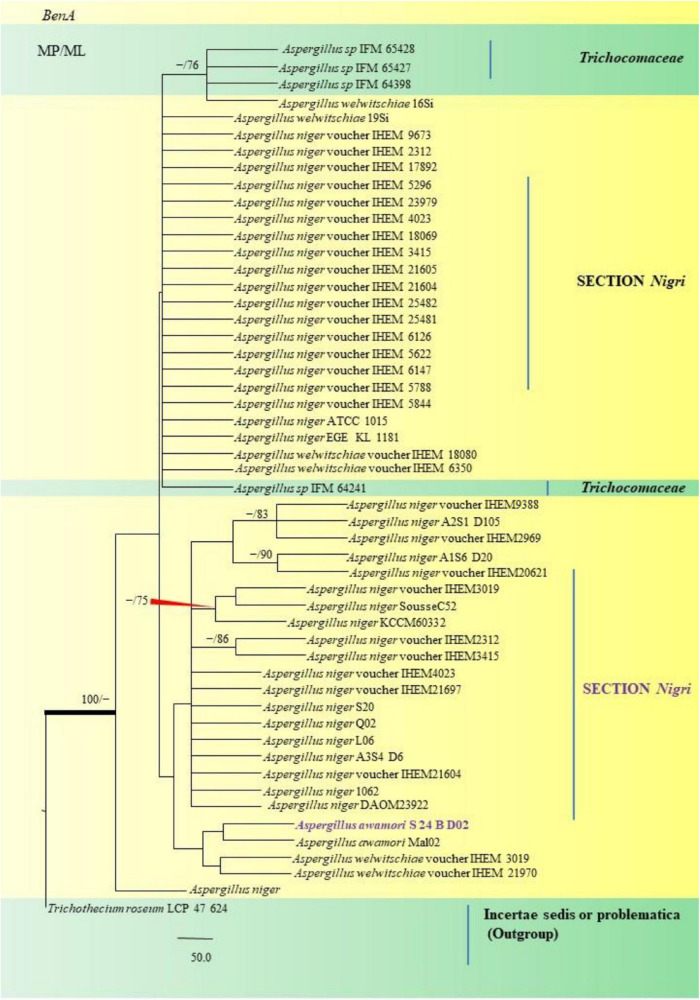
A phylogenetic tree (taxon branching diagram) which presents the genetic analysis of the *Aspergillus awamori* S 24 B D02 clade belonging to the Section *Nigri*, exhibiting a maximum parsimony analysis of partial β-tubulin concatenated sequences, acquired. Bootstrap (bs) values ≥ 70% are designated at the nodes for maximum parsimony (MP) and maximum likelihood (ML) methods; branches with values ≥ 95% bs are bold black. A specific isolate code was mentioned as a suffix to the species name. Hyphen (-) represents the range of support values for parsimony and RAxML analyses of below 70%. Our isolate, which is marked in purple, was rooted using *Trichothecium roseum* LCP 47 624.

The PCR products of the ITS (Amplicon size: 600-bp) and β-tubulin (Amplicon size: 400-bp) genetic markers were analyzed (Figures 6A,B). Based on sequence analysis of ITS 1, ITS 4 and β-tubulin genes, eleven (11) monophyletic groups of *Aspergilli* have been well resolved into six Sections: *Terrei*, *Flavi*, *Fumigati*, *Nidulantes, Nigri* and *Versicolores*. The ITS marker, which is the most suitable, profoundly recommended and widely accepted fungal barcode (Schoch et al., 2012) served handy in the recognition, identification, and classification of species in their respective clades. Both ITS regions and β-tubulin gene phylogenetic trees are depicted in the phylograms. Data creation and post-sequencing of *Aspergillus* genes were examined, with results showing that the β-tubulin-based phylogenetic trees had a greater level of precision than those generated by the ITS regions. Analysis of β-tubulin-based phylogenetic trees for our isolates namely *Aspergillus aureoterreus* (Figure 7), *Emericella quadrilineata* (Figure 8), *Aspergillus latus* (Figure 9), and *Aspergillus awamori* (Figure 10) showed higher resolution. In addition, ITS-based phylogenetic trees for *Aspergillus terreus*, *Aspergillus nidulans*, *Aspergillus sydowii*, and *Aspergillus* sp., as well as β-tubulin-based phylogenetic trees for isolates of *Aspergillus flavus*, *Aspergillus tamarii*, and *Aspergillus fumigatus*, were created.

Isolates studied in the present analysis, namely, *A*. *aureoterreus* FOEVPRB18 (Figure 7) was closely associated with the type strain *A*. *aureoterreus* CMV010F6 (MK451161), which has been deposited very recently at NCBI by researchers, while conducting studies to update the taxonomical status of *Aspergillus* species from South Africa (Visagie and Houbraken, 2020). The type strain *A*. *terreus* CVS503 65 (EU147717), was the most closely related species with our isolate (Figure 7), known to be a notorious fungus, playing a key role in human opportunistic pathogenesis, unpublished (Balajee et al., 2009). While *A. aureoterreus* CMV010F6 displayed 99.37% identity with our isolate *A. aureoterreus* FOEVPRB18, *A. terreus* CVS503 65 showed 96.32% identity.

**FIGURE 11:**
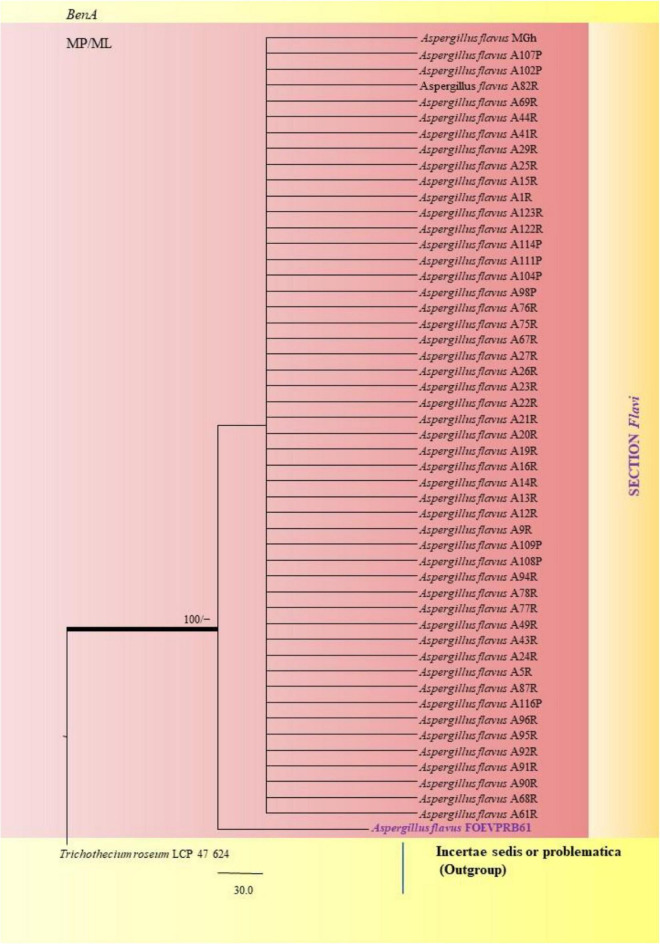
A phylogenetic tree (taxon branching diagram) which presents the genetic analysis of the *Aspergillus flavus* FOEVPRB61 clade belonging to the Section *Flavi*, exhibiting a maximum parsimony analysis of partial β-tubulin concatenated sequences, acquired. Bootstrap (bs) values ≥ 70% are designated at the nodes for maximum parsimony (MP) and maximum likelihood (ML) methods; branches with values ≥ 95% bs are bold black. A specific isolate code was mentioned as a suffix to the species name. Hyphen (-) represents the range of support values for parsimony and RAxML analyses of below 70%. Our isolate, which is marked in purple, was rooted using *Trichothecium roseum* LCP 47 624.

**FIGURE 12:**
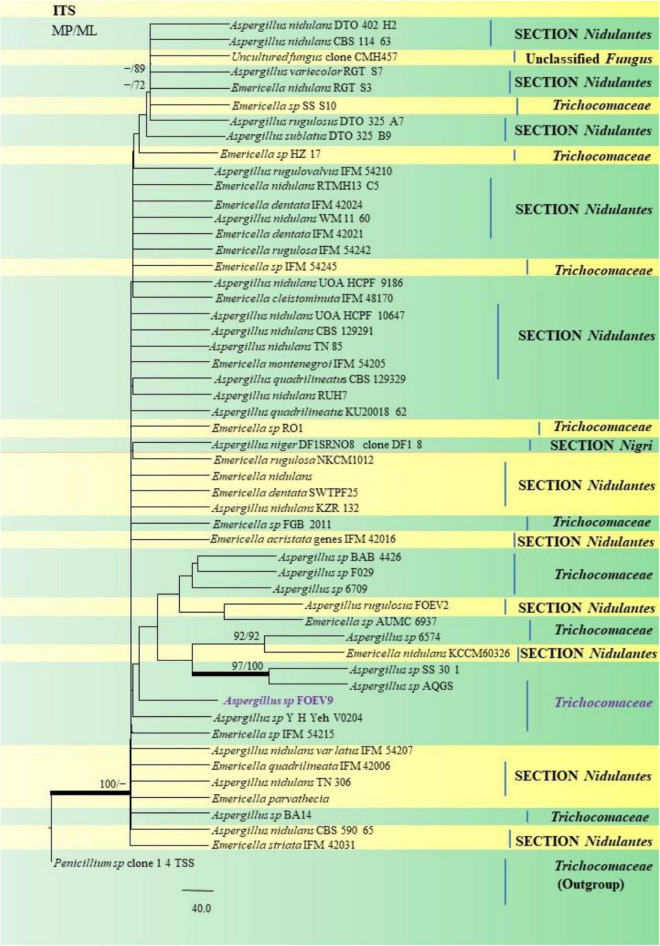
A phylogenetic tree (taxon branching diagram) which presents the genetic analysis of the *Aspergillus* sp. FOEV9 clade belonging to the family *Trichocomaceae*, exhibiting a maximum parsimony analysis of partial ITS 1 and ITS 4 concatenated sequences, acquired. Bootstrap (bs) values ≥ 70% are designated at the nodes for maximum parsimony (MP) and maximum likelihood (ML) methods; branches with values ≥ 95% bs are bold black. A specific isolate code was mentioned as a suffix to the species name. Hyphen (-) represents the range of support values for parsimony and RAxML analyses of below 70%. Our isolate, which is marked in purple, was rooted using *Penicillium species* clone 1 4 TSS.

Similarly, the clades *A*. *flavus* and *A*. *tamarii* were placed under the *Flavi* Section. *A*. *flavus* FOEVPRB61 occurred as a separate taxon although it was closely related to previously described clades of the same species (Figure 11). Partial ITS and β-tubulin gene sequences were used to arrive at species identity of the 27 *Aspergillus* isolates. Sequences from both the genetic markers gave almost the same identification. However, a doubtful analysis was experienced with *Aspergillus* isolate (ITS Barcode: MN294688; Section Unassigned), in determining a species name (Figure 12). Nevertheless, a detailed macro- and micro-morphological analysis of the unassigned fungal isolate is available. Although it emerged as a separate taxon during phylogenetic analysis, it showed 100% bs value (ML method) with *Aspergillus* sp. SS 30 1 studied by another research team from India, Direct Submission, Genetics, Agharkar Research Institute, Maharashtra, India (Chavan, 2020). This species was studied with *Penicillium* species clone 1 4 TSS as an outgroup. In most instances, our isolates were closely related to type strains which have been previously studied and described by providing appreciable support values.

**FIGURE 13:**
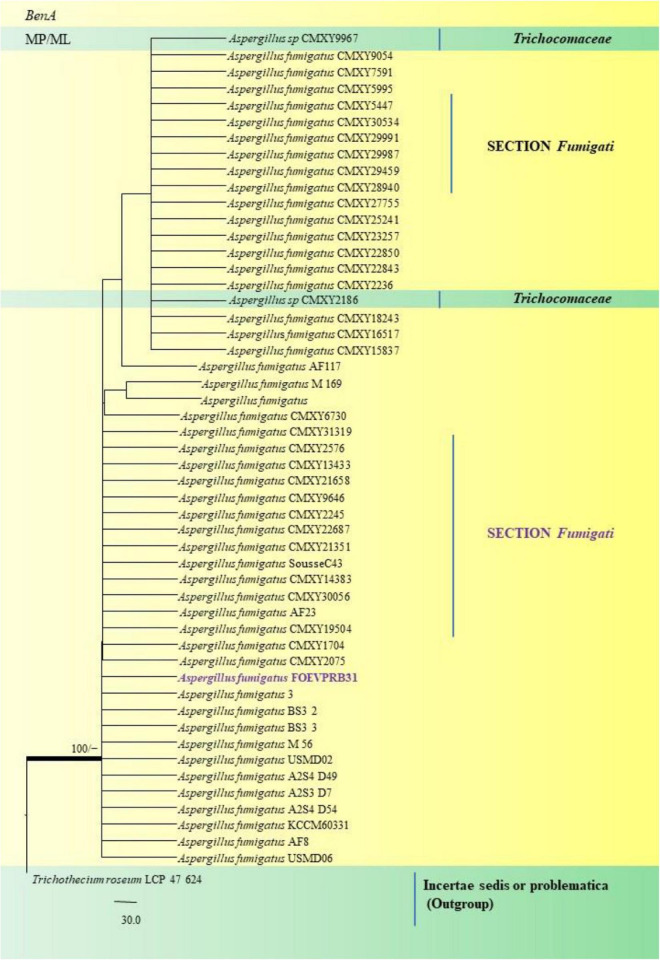
A phylogenetic tree (taxon branching diagram) which presents the genetic analysis of the *Aspergillus fumigatus*FOEVPRB31 clade belonging to the Section *Fumigati* exhibiting a maximum parsimony analysis of partial β-tubulin concatenated sequences, acquired. Bootstrap (bs) values ≥ 70% are designated at the nodes for maximum parsimony (MP) and maximum likelihood (ML) methods; branches with values ≥ 95% bs are bold black. A specific isolate code was mentioned as a suffix to the species name. Hyphen (-) represents the range of support values for parsimony and RAxML analyses of below 70%. Our isolate, which is marked in purple, was rooted using *Trichothecium roseum*LCP 47 624.

In the Section *Fumigati* our isolate namely *A. fumigatus* FOEVPRB31 (Figure 13) was found to be a close relative of *A. fumigatus* 3, Direct Submission, Department of Dermatology, Nanjing University, China (Chen et al., 2018) and *A. fumigatus* CMXY2075, Direct Submission, Ecology of Clinical Fungi, Fungal Biodiversity Institute, Netherlands (Chen and Xu, 2018).

**FIGURE 14:**
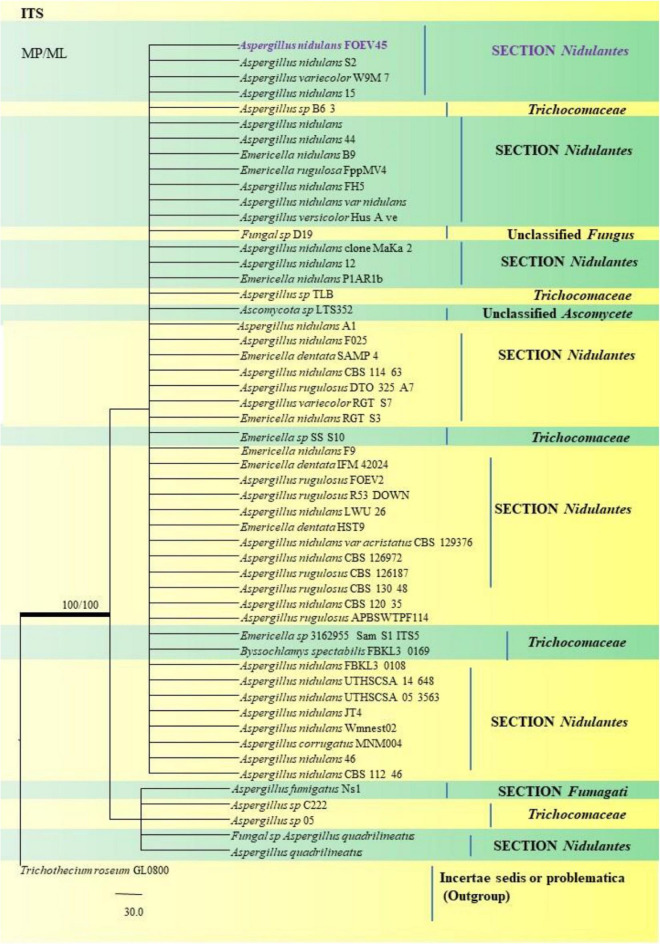
A phylogenetic tree (taxon branching diagram) which presents the genetic analysis of the *Aspergillus nidulans* FOEV45 clade belonging to the Section *Nidulantes*, exhibiting a maximum parsimony analysis of partial ITS 1 and ITS 4 concatenated sequences, acquired. Bootstrap (bs) values ≥ 70% are designated at the nodes for maximum parsimony (MP) and maximum likelihood (ML) methods; branches with values ≥ 95% bs are bold black. A specific isolate code was mentioned as a suffix to the species name. Hyphen (-) represents the range of support values for parsimony and RAxML analyses of below 70%. Our isolate, which is marked in purple, was rooted using *Trichothecium roseum* GL0800.

*A*. *nidulans*, *A*. *quadrilineatus* and *A*. *latus* were conveniently assigned in their clades under the *Nidulante*s Section. *A*. *nidulans* is phenotypically very similar to *A*. *quadrilineatus* except for differences in ascospore morphology (Chen et al., 2016). With respect to their phylogeny too they were situated in closely related clades, emerging from that of *E*. *nidulans* which is the sexual morph of *A*. *nidulans* (Figure 14). The sexual stages were appreciably documented in our isolate also, as realized by micro-morphological analyses. However, the concept of naming *Aspergillus* species with their sexual morphs is gradually becoming obsolete, due to major decisions taken at the meeting of the International Commission for *Penicillium* and *Aspergillus* (ICPA) in 2012.^6^ *A. quadrilineatus* and *A. latus*, which shared 99.76% identity with the type strain *Emericella quadrilineata* IFM 42006 (AB248335.1) and 99.55% identity with the type strain DTO 047 H2 (KU866810.1) respectively, were classified into well-defined clades with *A. quadrilineatus*, which formed discrete and conspicuous sexual stages and therefore is referred to as *Emericella quadrilineata* (Teleomorph). This was also in support of data generated through phylogenetic analysis of β-tubulin gene (Figure 8) being closely related to *Emericella quadrilineata* IFM 42006 (AB248335.1) studied previously (Matsuzawa et al., 2006). *A*. *latus* was found to emerge as a separate taxon (Figure 9) from *A*. *quadrilineatus* DTO 048 A8, Direct Submission, CBS-KNAW Fungal Biodiversity Centre (Chen et al., 2016) and displayed 100% bs value with *Emericella foveolata* IFM 54285 and *E. foveolata* IFM 42015 (Matsuzawa et al., 2006), which has also been studied under the Section *Nidulantes* while discerning evolutionary relatedness.

*Aspergillus awamori* S24 BD02 isolated from the fennel samples diverged as a clade closely related to *A*. *awamori* Mal02, Direct Submission, Department of Biotechnology, University of Verona, Italy (Andreolli et al., 2018), which in turn were related to *A*. *niger* studied collectively as black *Aspergilli*. Only one isolate namely *A*. *sydowii* FOEV83 was studied under the Section *Versicolores*. Studies inferred by phylogenetic analyses showed its close relatedness to *A*. *sydowii* DUCC5715, Direct Submission, Department of Microbiology, Dankook University, South Korea (Ahn et al., 2020), and a 100% bs value with *A*. *versicolor* M4 C4, Direct Submission, Postgraduate in Biological Sciences, University of Nayarit, Mexico (Bobadilla-Carrillo et al., 2020). This was especially true with cultural and morphological studies, where *A*. *sydowii* FOEV83 appeared very similar to *A*. *versicolor* by the characteristic pigmentation.

### Single Nucleotide Polymorphisms

In order to study the variants in the β-tubulin genes, the sequences obtained from Sanger sequencing were aligned to the reference genome sequences of respective species. The alignment was carried out using BLASTn algorithm. The variants such as single nucleotide variants, substitutions, insertions, and deletions were identified manually. Based on the regions of coding sequence (CDS) of β-tubulin gene provided at the protein feature annotations, the effect of these mutations at protein level were manually evaluated. The site of variations at the genome and protein level are tabulated (Table 2). Once these sequences for each species had been aligned, we observed that they all share a high degree of identity and displayed few variants with the exceptions of MN791095, MN791096, MN791109, and MN791093 sequences. We found that five species MN791107: *A. nidulans* 4, MN791105: *E. quadrilineata* 2, MN791112: *A. latus* 3, MN791108: *A. tamarii* 2, and MN791099: *A. fumigatus* 1 did not exhibit variations with regard to the reference genome in their respective sections. Hence, these five species have been excluded (Table 2). Our SNP analysis showed a high number of intronic variances and protein coding synonymous variants (changes without coding sequences) that result in synonymous mutations. On the contrary, four non-synonymous (substituting amino acids with new ones) variants were identified- MN791102: *A. nidulans* 3, MN791116: *E. nidulans* 5, MN791104: *E. quadrilineata* 1, and MN791110: *A. latus* 1, all contain single nucleotide variations (SNVs) that alter protein sequences whose functional consequences has not been studied.

**TABLE 2:** Sanger’s sequencing data to provide a list of protein-altering mutations.

Sl. No.	Species	Our sequence ID	Genome references	Reference genome position	Mutation variant	Nucleotide substitution	Protein ID	Protein alteration	Mutation site
1	*Aspergillus nidulans*	MN791097.1	AACD01000016.1	426,219	Insertion	17insT	EAA66300.1	Nil	Intronic
2	*Aspergillus nidulans*	MN791097.1	AACD01000016.1	426,091	SNV	146C > A	EAA66300.1	−	Intronic
3	*Aspergillus nidulans*	MN791097.1	AACD01000016.1	426,075	SNV	162T > A	EAA66300.1	−	Intronic
4	*Aspergillus nidulans*	MN791097.1	AACD01000016.1	426,027	SNV	210A > G	EAA66300.1	p.43Q > Q	Protein coding: Synonymous
5	*Aspergillus nidulans*	MN791097.1	AACD01000016.1	425,990	SNV	138T > G	EAA66300.1	−	Intronic
6	*Aspergillus nidulans*	MN791097.1	AACD01000016.1	425,839	Double substitution	290AC > TG	EAA66300.1	p.89N > M	Protein coding: Non-synonymous
7	*Aspergillus nidulans*	MN791101.1	AACD01000016.1	425,867	SNV	261T > C	EAA66300.1	p.79G > G	Protein coding: Synonymous
8	*Aspergillus nidulans*	MN791102.1	AACD01000016.1	426,218	Insertion	19insT	EAA66300.1	−	Intronic
9	*Aspergillus nidulans*	MN791102.1	AACD01000016.1	426,075	SNV	174T > A	EAA66300.1	−	Intronic
10	*Aspergillus nidulans*	MN791102.1	AACD01000016.1	426,091	SNV	148C > A	EAA66300.1	−	Intronic
11	*Aspergillus nidulans*	MN791102.1	AACD01000016.1	426,028	SNV	212A > G	EAA66300.1	p.38G > G	Protein coding: Synonymous
12	*Aspergillus nidulans*	MN791102.1	AACD01000016.1	425,792	SNV	447C > T	EAA66300.1	p.105H > Y	**Protein coding: Non-synonymous**
13	*Aspergillus nidulans*	MN791116.1	AACD01000016.1	426,238	Double substitution	109TT > AC	EAA66300.1	p.16I > N	Protein coding: Non-synonymous
14	*Aspergillus nidulans*	MN791116.1	AACD01000016.1	425,859	SNV	489C > T	EAA66300.1	p.82G > G	Protein coding: Synonymous
15	*Aspergillus nidulans*	MN791116.1	AACD01000016.1	426,218	Insertion	20insT	EAA66300.1	−	Intronic
16	*Aspergillus nidulans*	MN791116.1	AACD01000016.1	426,132	SNV	107T > C	EAA66300.1	p.30L > P	**Protein coding: Non-synonymous**
17	*Aspergillus nidulans*	MN791116.1	AACD01000016.1	426,075	SNV	164T > A	EAA66300.1	−	Intronic
18	*Aspergillus nidulans*	MN791116.1	AACD01000016.1	426,091	SNV	148C > A	EAA66300.1	−	Intronic
19	*Aspergillus nidulans*	MN791116.1	AACD01000016.1	426,021	SNV	218G > A	EAA66300.1	p,45E > E	Protein coding: Synonymous
20	*Aspergillus nidulans*	MN791116.1	AACD01000016.1	426,027	SNV	212A > C	EAA66300.1	p.43Q > Q	Protein coding: Synonymous
21	*Aspergillus nidulans*	MN791116.1	AACD01000016.1	425,970	SNV	269G > A	EAA66300.1	−	Intronic
22	*Aspergillus nidulans*	MN791116.1	AACD01000016.1	425,786	SNV	453A > T	EAA66300.1	p.107T > S	**Protein coding: Non-synonymous**
23	*Aspergillus quadrilineatus*	MN791104.1	JAAXYA010000001.1	628,223	SNV	107T > C	ABW72458.1	p.10L > P	**Protein coding: Non-synonymous**
24	*Aspergillus quadrilineatus*	MN791104.1	JAAXYA010000001.1	628,112	SNV	218G > A	ABW72458.1	−	Intronic
25	*Aspergillus quadrilineatus*	MN791104.1	JAAXYA010000001.1	628,061	SNV	269G > A	ABW72458.1	−	Intronic
26	*Aspergillus quadrilineatus*	MN791104.1	JAAXYA010000001.1	627,877	SNV	453A > T	−	−	−
27	*Aspergillus latus*	MN791110.1	VCRL01000022.1	334,420	SNV	422T > A	QJS39736.1	p.83G > G	Protein coding: Synonymous
28	*Aspergillus latus*	MN791110.1	VCRL01000022.1	334,409	SNV	433A > T	QJS39736.1	p.87N > I	**Protein coding: Non-synonymous**
29	*Aspergillus latus*	MN791111.1	VCRL01000022.1	334,764	SNV	78C > G	QJS39736.1	−	Intronic
30	*Aspergillus tamarii*	MN791098.1	ML738700.1	20,713	SNV	10C > T	KAE8158230.1	p.21W > W	Protein coding: Synonymous
31	*Aspergillus tamarii*	MN791115.1	ML738700.1	20,700	Double substitution	12TA > AC	KAE8158230.1	p.16I > N	Protein-coding: Non-synonymous
32	*Aspergillus fumigatus*	MN791100.1	NC_007194.1	2,849,872	SNV	6T > C	XP_752456.1	−	Non-protein coding
33	*Aspergillus fumigatus*	MN791103.1	NC_007194.1	2,849,872	SNV	6T > C	XP_752456.1	−	Non-protein coding
34	*Aspergillus fumigatus*	MN791103.1	NC_007194.1	2,849,337	SNV	541C > T	XP_752456.1	−	Intronic
35	*Aspergillus awamori*	MN791113.1	BDHI01000014.1	2,536,570	SNV	13T > C	GCB22540.1	−	Intronic
36	*Aspergillus awamori*	MN791113.1	BDHI01000014.1	2,536,901	SNV	344T > G	GCB22540.1	−	Intronic
37	*Aspergillus awamori*	MN791113.1	BDHI01000014.1	2,536,908	SNV	351A > T	GCB22540.1	−	Intronic
38	*Aspergillus awamori*	MN791114.1	BDHI01000014.1	2,536,570	SNV	13T > C	GCB22540.1	−	Intronic
39	*Aspergillus awamori*	MN791114.1	BDHI01000014.1	2,536,901	SNV	344T > G	GCB22540.1	−	Intronic
40	*Aspergillus awamori*	MN791114.1	BDHI01000014.1	2,536,908	SNV	351A > T	GCB22540.1	−	Intronic
41	*Aspergillus awamori*	MN791114.1	BDHI01000014.1	2,536,658	SNV	108T > A	GCB22540.1	−	Intronic

*Bold values refers to non-synonymous SNV mutations that we found. Such mutations may have an effect on the protein coding functional outcomes if any.*

The SNP profiling analyses revealed five mis-sense mutations in the CDS (coding DNA sequence) regions of beta-tubulin gene in four *Aspergillus* isolates. Histidine was replaced by tyrosine at the 105th position in *A*. *nidulans* MN791102, leucine to proline and threonine to serine at positions 30 and 107, respectively, in *E. nidulans* MN791116. In *E. quadrilineata* MN791104 leucine was replaced to proline at the 10th position. Furthermore, analysis of *A*. *latus* MN791110 indicated that the amino acid asparagine was substituted with isoleucine at the 87th position.

## Discussion

Spices occupy a very prominent role in the culinary preparations in India (Siruguri and Bhat, 2015). Based on the limited information and scientific evidence available through literature, fennel continues to be the most widely used herb in traditional medicine. *F*. *vulgare* is known to exhibit an array of pharmacological properties such as antimicrobial, antiviral, anti-inflammatory, anti-mutagenic, antipyretic, anti-spasmodic, anti-thrombotic, apoptotic, cardiovascular, chemomodulatory, anti-tumor, hepatoprotective, and hypoglycemic. Also, they’ve been linked to memory enhancement (Badgujar et al., 2014), which implies that they could be useful in the field of pharmaceutical biology for the development of new drugs to treat a wide range of ailments.

The use of spices in food has been practiced since time immemorial. Black pepper, cardamom, clove, coriander, cumin, ginger and other spices have been explored for their intrinsic antibacterial effects when used in foods, in addition to the flavors and aromas that they produce. In spite of the antibacterial compounds that they harbor, they are vulnerable to mycological deterioration, constantly being colonized by mycoflora like the *Aspergilli*, *Penicillia*, and *Fusaria* during pre- and post-harvest operations at the field.

Accurate identification of *Aspergillus* isolates up to the species level has become ecologically, epidemiologically, and pathologically significant because of the extensive damage that they cause in food crops (Battilani et al., 2016; Alshannaq and Yu, 2017), feed crops (Ráduly et al., 2020), and spices (Ali et al., 2015; Ssepuuya et al., 2018). Specific studies focused on mycological analyses have been conducted by research groups on spices like cumin, clove, black pepper, ginger, cardamom, coriander and cinnamon with dominancy of *Aspergillus flavus* and *A. niger*, with cumin being the most contaminated sample (Elshafie et al., 2002). Notable contamination by *A. flavus*, *A. fumigatus*, *A. alutaceus*, *A. niger*, and *A. sulphureus* has been observed in yet another study conducted using aniseed, rosemary and spice products (Ahene et al., 2011). Another research group (Hammami et al., 2014) examined fourteen distinct spice samples and reported the highest fungal contamination by *Aspergillus flavus*, *A. nomius*, and *A. niger*. The studies conducted using fennel seeds from India (Kulshrestha et al., 2014) have been sparse, with reports on *Aspergillus* contamination being investigated from fennel seeds, cumin and coriander seeds which are extensively used in culinary preparations in India. Two other research studies (Azzoune et al., 2015; Garcia et al., 2018) have been conducted in order to evaluate the mycotoxigenic potential of select *Aspergilli*, from nearly about two hundred spice samples of rosemary, fennel, cinnamon, clove, pepperoni, black and white pepper and oregano. Our study is the first of its kind to reveal a significant degree of *Aspergillus* contamination in the Indian subcontinent, with *A. niger* being the most frequently isolated species, followed by *Mycelia sterilia*, *A*. *flavus*, *Mucor* species, *Penicillium* species, and Yeasts, among others.

The *Aspergilli* are an all-pervasive and species substantial genus, containing more than three hundred thread-like filiform fungi (Anonymous, 2021). By far, the majority of *Aspergilli* from *F. vulgare* were found in the *Nigri* section in this study, which was collected from distinct geographical locations across India, including Delhi, Pondicherry, Rajasthan, Uttar Pradesh, and West Bengal. Isolates studied here, are predominantly predisposed with a toxigenic potential, known to cause a wide range of human and animal diseases, in addition to being plant pathogens.

Two *Aspergilli*, namely *Aspergillus aureoterreus* and *A. terreus*, were isolated from fennel samples classified under the *Terrei* Section. *A. aureoterreus* differs from *Aspergillus terreus* by the sequence analyses of the regions of the beta-tubulin genes (Samson et al., 2011). In the phylogenetic studies, the *A. aureoterreus* species formed a distinct lineage different from the *A. terreus* clade (Samson et al., 2011). The type-strain was isolated from *Foeniculum vulgare* (Sample No.18, place-Midnapore, West Bengal) and has been deposited in the National Center for Biotechnology Information (NCBI) GenBank with Accession No. MN791096.

*A. sydowii* has also been recognized as *Emericella sydowii*, a soil fungus, that could adulterate foodstuffs. It is also a pathogen known to cause human diseases like aspergillosis, onychomycosis, and keratomycosis (de Hoog et al., 2000). Apart from its disease-causing and toxigenic potential, *A. sydowii* has been the source of anticancer alkaloids. This species generates alkaloids, namely, 6-methoxyspirotryprostatin B, 18-oxotryprostatin A and 14-hydroxyterezine D (Zhang et al., 2008) and have potential cytotoxic activity against a549 cells (Nadumane et al., 2016).

*Aspergillus flavus* has received continual attention ever since its aflatoxigenic potential was discovered. The mold placed under the *Flavi* section, is known to be air-borne, thermotolerant and popularly as a saprophytic soil fungus. The fungus has been found all over the world (Ramírez-Camejo et al., 2012) and is abundant in temperate soil (Thom and Raper, 1945). *A*. *flavus* was found to be the most frequently occurring species in the fennel samples analyzed in the present study after *A*. *niger* and *Mycelia sterilia*, is known to be highly infectious with a broad range of infectivity and is able to contaminate agricultural crops during pre-harvest and post-harvest procedures (Bignell, 2010). In certain regions in Africa and Asia, AFs are appraised to be a major cause of severe acute sickness and loss of several lives per year (Wood, 1992; Alshannaq and Yu, 2017). The fungus is the most common source of aflatoxins in contaminated agricultural crops, can grow well in order to release aflatoxins frequently in almost any crop seed. The aflatoxins (AFs) are highly carcinogenic and immunosuppressive in nature, that has threatened global food safety, and has proven to be hazardous to animals, insects, and humans. It is an opportunistic fungal pathogen to humans as well as animals causing aspergillosis in immune-compromised hosts. In mammals, the pathogen can induce cancer of the liver (Amaike and Keller, 2011). Globally, over 4.5 billion people have been exposed to unmonitored levels of AFs (Alshannaq et al., 2018). Aflatoxin B_1_ (AFB_1_) is a highly toxic and the most potent natural compound to cause hepatocellular carcinoma in the universe. AFs are severely toxic, mutagenic, carcinogenic, teratogenic, immunosuppressants and are classified as Group1 carcinogens in human beings (Ostry et al., 2017) and hence are of special concern to human, animal and poultry health. *A. tamarii* is an epidemiological agent of human mycotic keratitis in India (Homa et al., 2019). Although it does not produce aflatoxins, it can induce elaboration of other toxic compounds like cyclopiazonic acid (Dorner et al., 1983; Ito, 1998), hence, posing a threat to human health.

*Aspergillus fumigatus* was studied under the *Fumigati* section and was isolated from fennel samples. With regard to the formation of sexual structures, sclerotia were not produced in the isolates studied by us. *A. fumigatus* has long been thought of reproduce only by asexual means until a detailed study was published by two researchers (Dyer and Paoletti, 2005). They elaborated on the possibility of the occurrence of sexual reproduction in *A. fumigatus*. Also, the detailed investigations and opinions of several mycologists have led to the findings, that point toward evidence that sexual reproduction may occur within populations of *A. fumigatus*. Teleomorphs of *A. fumigatus* are yet to be detected. However, it would be very significant to note that, the presence of sexual cycles would lead to an impending understanding of their biology and life cycles (Dyer and Paoletti, 2005). Other perspectives to understanding this species is its role as a human pathogen. *A. fumigatus* has been the highest prevailing aerial fungal parasite in immunodeficient hosts and is known to be associated with lethal systemic septicemia in multiple organs. It causes incursive infection in the lungs or long-term infection affecting the lungs, hypersensitive bronchopneumonia aspergillosis or allergic disorders in immune deficiencies (Hohl and Feldmesser, 2007; Segal, 2009). The disease can spread especially in individuals suffering from certain types of leukemia (Parahym et al., 2014) and those who are at the advanced stages of AIDS (Kaur et al., 2017), ailing with bronchial asthma (Kosmidis and Denning, 2015), fibrocystic disease of the pancreas (Düesberg et al., 2020), and in patients who have recovered from an influenza infection (Van De Veerdonk et al., 2017).

*A. nidulans* is a deuteromycetous member, which falls under the category of polyphyletic group of fungi whose sexual stages (teleomorph) are rare or unknown. In spite of this, mutants of *A. nidulans* are known to produce copious amounts of Hülle cells in the absence of sexual stages (Dyer and O’Gorman, 2012). A large number of species in the section *Nidulantes* have a sexual state whose name is *Emericella*, in the dual nomenclature system of fungi. If sexual stages are known and observed, the teleomorph has taxonomic precedence over the asexual stage, generally referred to as anamorph. Therefore, this species is most properly known as *Emericella nidulans*. Nevertheless, this fungal species will continue to be known in literature and the scientific or commercial industry for its inclusiveness as *A. nidulans*, which may be used here for both the anamorph and teleomorph interconvertibly. Asci contained eight-spored cells ascospores which were reddish/cherry-brown and lens-shaped and correlated with the original description by Thom and Raper (1945). A detailed microscopic description is available (Chen et al., 2016) for ascospore morphology of *Aspergilli* of the *Nidulantes* section. The ascospore color, ornamentation, shape and size are of particular value for differentiating species.

*A. latus* and *A*. *quadrilineatus* are being reported from *F*. *vulgare* for the very first time. Also, *A*. *latus* has been found to be phylogenetically similar to *A. sublatus* and *A. montenegroi*, thus considered synonymous. Ascospore morphology, which is a major and distinguishing feature while studying the sexual stages of different *Aspergilli*, were also documented in the current micromorphological analyses and were found to be orange/reddish brown with two pleated equatorial crests as studied recently and reported by Chen et al. (2016). The ascospores usually have two equatorial crests, whereas four crests can be seen in *A*. *quadrilineatus* by the aid of Scanning Electron Microscopy (SEM) and can sometimes be low or inconspicuous (Hubka et al., 2016). It has also been opined by Chen et al. (2016) that *A*. *nidulans* resembles *A*. *quadrilineatus* and is distinguishable in terms of its ascospore morphology. Phylogenetically *A*. *miyajii* has been found to be identical with *A*. *quadrilineatus*, *A*. *parvathecius A*. *acristatus*, and *A*. *floriformis* (Chen et al., 2016). *A. awamori* is being reported for the first and foremost time in fennel samples and is regarded as a domesticated form of *A*. *niger* (Samson et al., 2006) and its economic importance in Koji fermentation has found to be noteworthy (Kitamoto, 2002).

Nevertheless, the fungal phylogeny of *Aspergillus* species has undergone radical modifications over the course of over a hundred years. Phylogenetic analysis was especially useful when it was used to authenticate our morphological identification. In most instances, morphological identification and phylogenetic analyses were in agreement and helped in ascertaining species identity in *A*. *awamori* from the *Nigri* Section because of the striking phenotypic similarities to *A*. *niger* exhibited by our isolates. Phylogenetic studies were particularly useful while studying *A. aureoterreus*, *A. latus* and *Emericella quadrilineata* from Sections *Terrei*, and *Nidulantes*, respectively, along with *A*. *awamori*, which are being distinctly reported for the first time in *F*. *vulgare*. As a result, we were able to successfully distinguish them from closely related species and identify their Sections using the two genetic markers, which was precisely what we set out to do when this research was conceived.

**FIGURE 15:**
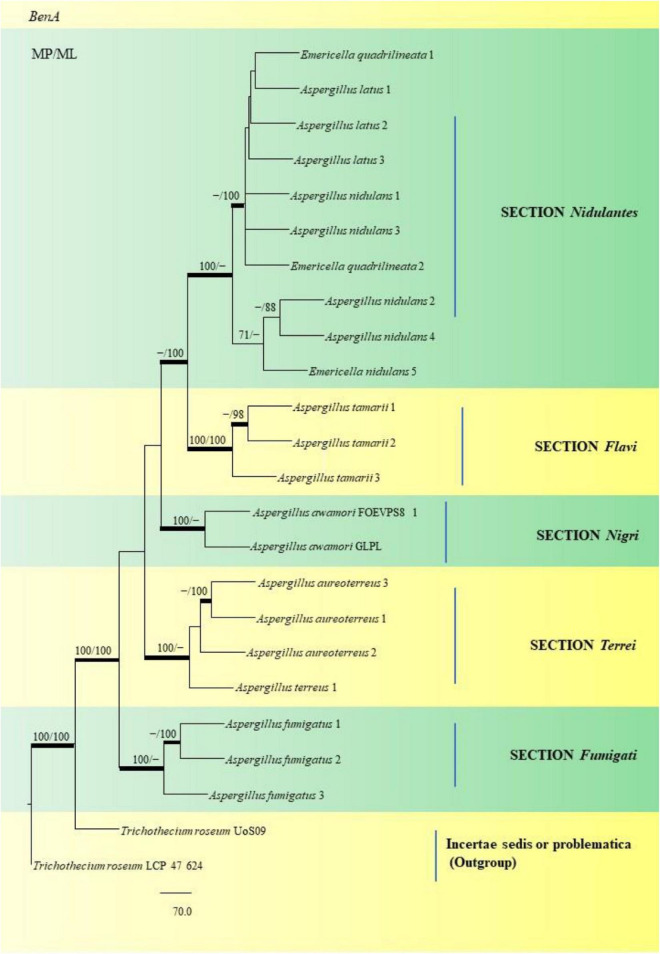
This phylogram depicts the beta-tubulin phylogenetic tree of 22 *Aspergillus species* in sections, and subsequent analysis of their single nucleotide polymorphisms is discussed below. Maximum parsimony analysis was used to create this tree, which incorporates partial β-tubulin concatenated sequences. Boot strap (bs) values ≥ 70% are designated at the nodes for maximum parsimony (MP) and maximum likelihood (ML) methods; branches with values ≥ 95% bs are bold black. Hyphen (-) indicates support values below 70% for both parsimony and RAxML analyses. *Trichothecium roseum* Uo S09 and *Trichothecium roseum* LCP 47 624 were used to root the isolates. The Fungal Genetics and Mycotoxicology (FGM) laboratory codes for the isolates are listed here. 49: *E. quadrilineata* 1, 4: *A. latus* 1, 11: *A. latus* 2, 12: *A. latus* 3: 17: *A. nidulans* 1, 9: *A. nidulans* 3, 58: *E. quadrilineata* 2, 45: *A. nidulans* 2, 71: *A. nidulans* 4, 2: *E. quadrilineata*, 22: *A. tamarii* 1, 75: *A. tamarii* 2, S44: *A. tamarii* 3, S8: *A. awamori* S8, S24: *A. awamori* GLPL, 51: *A. aureoterreus* 3, 18: *A. aureoterreus* 1, 1: *A. aureoterreus* 2, 16: *A. terreus* 1, 24: *A. fumigatus* 1, 37: *A. fumigatus* 2 and 31: *A. fumigatus* 3.

Through phylogenetic analysis and tree construction, we were able to study twenty-two (22) *Aspergillus* species for the SNP analysis (Figure 15), classifying them into the Sections *Nidulantes*, *Flavi*, *Nigri*, *Terrei*, and *Fumigati*. Under the *Nidulantes* section, we could classify two *A. latus* (Isolate Nos. 1 and 2), five *A. nidulans* (Isolate Nos. 1, 2, 3, 4 and 5) and two *E. quadrilineata* (*E. quadrilineata* Isolate No. 1 and *E. quadrilineata* Isolate No. 2). Thus, the species of *E. quadrilineata* (Isolate No. 1) is rather closely connected to *A. latus* (Isolate No. 1) on one end, and *A. nidulans* (Isolate No. 3) is closely related to *E. quadrilineata* (Isolate No. 2) on the other end. *F. vulgare* samples from Pondicherry showed the presence of *E. quadrilineata* (Isolate No. 1) and *A. latus* (Isolate No. 1), and *A. nidulans* (Isolate No. 3) and *E. quadrilineata* (Isolate No. 2) were obtained from Rajasthan. While speaking about the diversity of fungi in the *F. vulgare* samples, three isolates of *A. latus* were studied: *A. latus* (Isolate Nos. 1 and 2) were obtained from Pondicherry, while *A. latus* (Isolate No. 3) was isolated from Jhargram, West Bengal, India. In the case of the occurrence of *A. nidulans* species, *A. nidulans* (Isolate Nos. 1, 2, 4, and 5) were from Pondicherry, whereas *A. nidulans* (Isolate No. 3) had been obtained from Rajasthan. This phylogenetic tree (Figure 15) of *Aspergilli* from *F. vulgare* samples from several regions demonstrate how closely related strains are distributed geographically. Additionally, in the section *Flavi*, three *A. tamarii* species appear, two of which, specifically (*A. tamarii* Isolate Nos. 1 and 2) were found to be more closely related and were isolated from Pondicherry spice samples. The *A. tamarii* (Isolate No. 3) from New Delhi emerged as a separate clade. Under the *Nigri* section, *A. awamori* species has been studied: *A. awamori* (FOEVPS8 1) was isolated from Pondicherry, while *A. awamori* (GLPL) was isolated from West Bengal. Three *A. aureoterreus* species and one *A. terreus* were grouped in the *Terrei* section. *A. aureoterreus* (Isolate No. 1) and *A. terreus* (Isolate No. 1) was recovered from a *F. vulgare* sample collected from New Delhi, *A. aureoterreus* (Isolate Nos. 2 and 3) were isolated from Pondicherry fennel seeds. *A. fumigatus* (Isolate Nos. 1 and 2) from Section *Fumigati*, were isolated from a Pondicherry fennel sample, whereas *A. fumigatus* (Isolate No. 3) which emerged as a separate branch was obtained from Lucknow. Strains are likely to represent a diverse set of features within the section under investigation because of the divergence between strains within clades.

The SNV analysis reveals that several mutations were identified exclusively in the *Nidulantes* section. Strain *E. nidulans* 5 displayed most SNPs (Figure 15) suggesting that the *Aspergilli* identified under this section may contain an isolate that is distinct. This further divulges information that, there was considerable genetic heterogeneity between *A. nidulans*, *E. quadrilineata* and *A. latus* species. Additionally, the genetic divergence between distant isolates supports distribution of *Aspergilli* across geographic regions. Furthermore, as demonstrated in the *Flavi*, *Fumigati*, and *Nigri* sections, there is no variance since the genetic sequences employed as markers in this study are highly conserved in terms of SNV mutations, showing that most SNPs have a small effect on the protein structure. The non-synonymous SNPs that were identified in our study, may have significant favorable or negative outcomes and needs to be investigated. The variants may be unique and could be used as biological markers to study specific species. However, the presence of SNPs in *Aspergillus* species may help us understand the rationale behind genetic diversity with implications of functional consequences if any, through further explorations on their mycotoxigenic potential and related prospective studies. According to the present study, the highlights include *Aspergillus* diversity from fennel seeds, a polyphasic approach to reappraise *Aspergilli* into six sections using the official fungal barcode and functional genetic markers which have been performed in adequate detail, with β-tubulin coding sequences being the focus for SNP analysis. Substitutions, insertions and SNV-kind of mutations have been identified among the *Aspergillus* isolates considered in this study. The data represented is the first report to the best of our knowledge. The occurrence of *Aspergilli* may help us determine the health status of the spices or cereals as indicator organisms, to adopt suitable strategies for avoidance of contamination at the field and during storage.

## Conclusion

The study was an elaborate investigation on the isolation and phenotypic characterization of *Aspergilli* from an Indian spice namely *F*. *vulgare*. While nearly 27 isolates have been adequately described with substantive morphological descriptions, attempts have been made to characterize these *Aspergilli* phylogenetically as well. A key strength of this endeavor is the number of fennel samples analyzed and *Aspergilli* being reported in a “first of it’ s kind” investigation. The research study constitutes a key milestone in analyses of evolutionary relationships of fungi, detection of single nucleotide polymorphisms in the target fungal isolates. This validates genetic information, which serves as an essentiality for existing and further research. Although, the study may have its limitations, we propose to expand on the number of isolates to be studied for SNPs and their possible impacts on protein structure and functional consequences. Studies on their mycotoxigenic potentials is currently underway. Clearly, with respect to the diversity of *Aspergillus* species *F*. *vulgare*, the study is the largest till date. The extensive use of partial beta-tubulin gene analyses to evaluate the association between SNPs in five *Aspergillus* species sections is one of the highlights. Our research outcomes suggest that the presence of *Aspergilli* in food stuffs may pose a considerable hazard and threat to human consumption. Hence, identification and characterization may serve as a key factor in designing strategies to control post-harvest contamination and elaboration of mycotoxins. Fungal genomics serves as a useful molecular tool for inquiries into fungal evolution, by detecting gene differences and gene structure, to deduce the genetic basis of fungal evolution. However, blending morphological characteristics with molecular phylogeny is considered equally important for taxonomic studies and is the norm today.

## Data Availability Statement

The datasets presented in this study can be found in online repositories. The names of the repository/repositories and accession number(s) can be found below: https://www.ncbi.nlm.nih.gov/genbank/, MN791093; https://www.ncbi.nlm.nih.gov/genbank/, MN791096; https://www.ncbi.nlm.nih.gov/genbank/, MN791109; https://www.ncbi.nlm.nih.gov/genbank/, MN791095; https://www.ncbi.nlm.nih.gov/genbank/, MN791106; https://www.ncbi.nlm.nih.gov/genbank/, MN791098; https://www.ncbi.nlm.nih.gov/genbank/, MN791108; https://www.ncbi.nlm.nih.gov/genbank/, MN791115; https://www.ncbi.nlm.nih.gov/genbank/, MN791099; https://www.ncbi.nlm.nih.gov/genbank/, MN791100; https://www.ncbi.nlm.nih.gov/genbank/, MN791103; https://www.ncbi.nlm.nih.gov/genbank/, MN791097; https://www.ncbi.nlm.nih.gov/genbank/, MN791101; https://www.ncbi.nlm.nih.gov/genbank/, MN791102; https://www.ncbi.nlm.nih.gov/genbank/, MN791107; https://www.ncbi.nlm.nih.gov/genbank/, MN791116; https://www.ncbi.nlm.nih.gov/genbank/, MN791104; https://www.ncbi.nlm.nih.gov/genbank/, MN791105; https://www.ncbi.nlm.nih.gov/genbank/, MN791110; https://www.ncbi.nlm.nih.gov/genbank/, MN791111; https://www.ncbi.nlm.nih.gov/genbank/, MN791112; https://www.ncbi.nlm.nih.gov/genbank/, MN791113; https://www.ncbi.nlm.nih.gov/genbank/, MN791114; https://www.ncbi.nlm.nih.gov/genbank/, MN298848; https://www.ncbi.nlm.nih.gov/genbank/, MN309877; https://www.ncbi.nlm.nih.gov/genbank/, MN264637; https://www.ncbi.nlm.nih.gov/genbank/, MN326529; and https://www.ncbi.nlm.nih.gov/genbank/, MN39290.

## Author Contributions

PM and RD engaged in the research project’s planning and design. PM carried out all the experiments, documented the findings, and wrote the manuscript. RD authored the results, drafted the manuscript, corrected the errors, and assisted in writing the manuscript. AP undertook the phylogenetic analysis, while BM performed the SNP analysis. All authors contributed to the article and approved the submitted version.

## Conflict of Interest

BM was employed by the International Tech Park. The remaining authors declare that the research was conducted in the absence of any commercial or financial relationships that could be construed as a potential conflict of interest.

## Publisher’s Note

All claims expressed in this article are solely those of the authors and do not necessarily represent those of their affiliated organizations, or those of the publisher, the editors and the reviewers. Any product that may be evaluated in this article, or claim that may be made by its manufacturer, is not guaranteed or endorsed by the publisher.

## Footnotes

1
http://doua.prabi.fr/software/cap3

2
https://www.ncbi.nlm.nih.gov/genbank/

3
https://blast.ncbi.nlm.nih.gov/Blast.cgi

4
https://mafft.cbrc.jp/alignment/server/

5
https://www.phylo.org

6
http://www.aspergilluspenicillium.org/
